# Spatio-temporal dynamics of microglia phenotype in human and murine cSVD: impact of acute and chronic hypertensive states

**DOI:** 10.1186/s40478-023-01672-0

**Published:** 2023-12-19

**Authors:** Lorena Morton, Philipp Arndt, Alejandra P. Garza, Solveig Henneicke, Hendrik Mattern, Marilyn Gonzalez, Alexander Dityatev, Deniz Yilmazer-Hanke, Stefanie Schreiber, Ildiko R. Dunay

**Affiliations:** 1 Institute of Inflammation and Neurodegeneration, Medical Faculty, Health Campus Immunology, Infectiology, and Inflammation (GC-I3), Otto-von-Guericke University, Leipziger Straße 44, 39120 Magdeburg, Germany; 2 Department of Neurology, Medical Faculty, Otto-von-Guericke University Magdeburg, Magdeburg, Germany; 3 German Center for Neurodegenerative Diseases (DZNE) Helmholtz Association, Magdeburg, Germany; 4 Faculty of Natural Sciences, Biomedical Magnetic Resonance, Otto-von-Guericke University, Magdeburg, Germany; 5 Medical Faculty, Otto-von-Guericke University, Magdeburg, Germany; 6 Center for Behavioral Brain Sciences (CBBS), Magdeburg, Germany; 7 Clinical Neuroanatomy, Department of Neurology, Institute for Biomedical Research, Ulm University, Ulm, Germany; 8 Center for Intervention and Research on Adaptive and Maladaptive Brain Circuits Underlying Mental Health (C-I-R-C), Jena-Magdeburg-Halle, Germany

**Keywords:** Chronic arterial hypertension, Cerebral small vessel disease (cSVD), Microglia, Spontaneously hypertensive stroke-prone rat (SHRSP)

## Abstract

Vascular risk factors such as chronic hypertension are well-established major modifiable factors for the development of cerebral small vessel disease (cSVD). In the present study, our focus was the investigation of cSVD-related phenotypic changes in microglia in human disease and in the spontaneously hypertensive stroke-prone rat (SHRSP) model of cSVD. Our examination of cortical microglia in human post-mortem cSVD cortical tissue revealed distinct morphological microglial features specific to cSVD. We identified enlarged somata, an increase in the territory occupied by thickened microglial processes, and an expansion in the number of vascular-associated microglia. In parallel, we characterized microglia in a rodent model of hypertensive cSVD along different durations of arterial hypertension, i.e., early chronic and late chronic hypertension. Microglial somata were already enlarged in early hypertension. In contrast, at late-stage chronic hypertension, they further exhibited elongated branches, thickened processes, and a reduced ramification index, mirroring the findings in human cSVD. An unbiased multidimensional flow cytometric analysis revealed phenotypic heterogeneity among microglia cells within the hippocampus and cortex. At early-stage hypertension, hippocampal microglia exhibited upregulated CD11b/c, P2Y12R, CD200R, and CD86 surface expression. Detailed analysis of cell subpopulations revealed a unique microglial subset expressing CD11b/c, CD163, and CD86 exclusively in early hypertension. Notably, even at early-stage hypertension, microglia displayed a higher association with cerebral blood vessels. We identified several profound clusters of microglia expressing distinct marker profiles at late chronic hypertensive states. In summary, our findings demonstrate a higher vulnerability of the hippocampus, stage-specific microglial signatures based on morphological features, and cell surface protein expression in response to chronic arterial hypertension. These results indicate the diversity within microglia sub-populations and implicate the subtle involvement of microglia in cSVD pathogenesis.

**Supplementary Information:**

The online version contains supplementary material available at 10.1186/s40478-023-01672-0.

## Introduction

Chronic arterial hypertension is a significant risk factor for all-cause dementia, including neurodegenerative Alzheimer’s disease (AD) and vascular cognitive impairment (VCI). Cerebral small vessel disease (cSVD), attributed to arterial hypertension, is primarily characterized by arteriolosclerosis and responsible for the development of VCI, and is associated with severe intracerebral hemorrhages [1–4]. Current cSVD diagnosis relies on downstream, mostly irreversible, brain pathologies such as lacunes, white matter hyperintensities (WMH), or hemorrhages [4]. Still, early microvascular dysfunction, specifically arteriolosclerosis, is suggested to play a pivotal role, marked by chronic low-grade oxidative microvascular injury, with consequent structural and functional adaptations such as blood–brain barrier (BBB) disruption and neuroinflammation [5–7]. Corroborative evidence from autopsy and molecular imaging studies supports the emergence of neuroinflammation as a major contributor to the pathogenesis of hypertension-related arteriolosclerosis and cSVD [8–14]. As neuroinflammation also plays a major role in AD pathogenesis, this phenomenon may be a critical link between neurovascular and neurodegenerative diseases [15].

Microglia, primary contributors to neuroinflammation [16–18], respond to peripheral low-grade inflammation by disrupting the BBB and facilitating leukocyte infiltration, as it has been shown in murine models developing systemic inflammation, autoimmune diseases [19, 20] and human low-grade inflammation attributed to elevated arterial hypertension [21]. This transduction of inflammation, characterized by pro-inflammatory cytokines and chemokines commonly found in AD and VCI, including cSVD, can also induce adhesion molecule expression on brain endothelial cells, leading to leukocyte infiltration, synaptic pruning, and demyelination [22–24]. In hypertensive cSVD, regions of increased microglial reactivity have been observed, suggesting their involvement [12]. However, the spatial, temporal, and causal relationship between microglial reactivity, BBB leakage, and peripheral inflammation remains unclear. Recent evidence highlights the diversity of microglia throughout life, with a high heterogeneity during early development, a more homogenous population in adulthood, and a re-emergence of heterogeneity during senescence, which may play a role in pathological conditions [25–28]. Here, we investigated microglial features in the Spontaneously Hypertensive Stroke-Prone Rat (SHRSP), a rodent model of hypertensive cSVD [29–32], and in human post-mortem brain tissue with cSVD. Our study focuses on morphological features and vascular-associated microglia (VAMs) in both the SHRSP model and human cSVD. We aim to understand the role of microglia in cSVD progression, including early- and late-stage arterial hypertension and how microglial morphology relates with hypertension-induced surface marker expression in the SHRSP model.

## Materials and methods

### Human subjects

9 human autopsy cases were included and control cases were randomly selected within the same age range as the cSVD cases. Brains of all subjects underwent routine neuropathological examination and were screened for tauopathies, alpha-synucleinopathies and beta-amyloid (Aβ) deposition. Inclusion criteria for cSVD cases were pathologically confirmed white matter lesions (WML), and exclusion criteria included neurodegenerative disorders; i.e., Alzheimer-related tau pathology exceeding Braak’s neurofibrillary stage II [33] or other tauopathy, Parkinson’s disease except incidental subcortical Lewy bodies in the lower brain stem (stage < 3) according to Braak et al. [34], multisystem atrophy or other alpha-synuclein-related pathology, and presence of cerebral amyloid angiopathy (CAA). Altogether 4 cases with cSVD (3 females, 1 male) and 5 control cases (3 females, 2 males) were investigated. The age range of the two groups presented as Mean ± SD were 64.0 ± 16.0 in the cSVD group and 63.7 ± 12.7 in the control group. Further demographics and relevant data for the patient cohort are provided in Additional file 1: Table S1. This retrospective study was performed in compliance with the University Ethics Committee guidelines and German federal and state law governing human tissue usage. Informed written permission was obtained from all patients and/or their next of kin for autopsy.

### Animals

All experiments adhered to the German Animal Welfare Ordinance and were approved by the Animal Care Committee of Saxony-Anhalt (license identification number 42502-2-1561 Uni MD). 32 male SHRSP rats (Charles River Laboratories, Wilmington, Massachusetts, USA) (25 and 34 weeks old) and 32 male age-matched Wistar rats (Charles River Laboratories, Research Models and Services, Germany GmbH, Sulzfeld, Germany) we used. From this point forward, Wistar rats are designated as controls, and SHRSP rats as early (25 weeks) and late (34 weeks) chronic hypertensive rats. All animals were housed with a natural light-night cycle, had access to water and food ad libitum and were monitored daily to assess neurological functions. SHRSP rats develop a vascular risk profile characterized by arterial hypertension between 6–8 weeks of age [32, 35–37]. Animals were neurologically unremarkable during the observation period. Additional file 1: Table S2 provides a concise summary of the methodologies employed.

### Immunofluorescence in rodent tissue

Animals were transcardially perfused under pentobarbital anesthesia (40 mg/kg body weight i.p.) with 120 mL of phosphate buffered saline, followed by perfusion with 120 mL of the fixative 4% paraformaldehyde (PFA). Brains were removed, immersion-fixed in 4% PFA for 48 h, cryoprotected in 30% sucrose for 6 days, and frozen in methyl butane at − 80 °C. Using a cryostat, brains were sectioned from the frontal to the occipital pole, and 30 µm-thick free-floating sections were collected in PBS. Immunofluorescence staining was performed as previously described [32, 38]. Four coronal brain sections were stained: (i) two containing the medial prefrontal and motor cortex (Bregma 4.7 to 2.5), and another two containing the hippocampus and retrosplenial cortex of both hemispheres per animal (Bregma − 2.5 to − 4.5 [39]). Sections were repeatedly washed in phosphate-buffered saline (PBS), blocked with 10% donkey serum/0.5% Triton X-100 (Sigma, St Louis, MO, USA), and incubated overnight at 4 °C with STL-FITC (endothelial marker, 1:750, solanum tuberosum lectin, Vector Laboratories FL-1161) and goat anti-IBA1 (microglia marker, 1:1500, ionized calcium-binding adapter molecule 1, Novus Biologicals NB100-1028). Sections were incubated for two hours the following day with anti-rat IgG Cy3 (BBB breakdown marker, 1:600, Immuno Research, 712–165-150) and anti-goat Cy5-conjugated secondary antibodies (1:500, Jackson ImmunoResearch, 703-175-155) and mounted on slides with Fluoromount Aqueos Mounting Medium (Merck, F4680).

### Image acquisition and analysis in the SHRSP model

A total of 20 animals were included, with 5 SHRSP assigned to early, 5 SHRSP for late chronic hypertension, and 5 age-matched normotensive controls for each stage. Images were acquired using a Zeiss confocal microscope (LSM 700). Z-stack images were acquired taken from 10 fields of view (FOV) per animal with a voxel size of 1.25 × 1.25 × 1 µm^3^ (for overall microglia soma counting, size quantification, and blood vessel association) and 4 fields of view per animal per region with voxel size of 0.16 × 0.16 × 0.3 µm^3^ (for single-cell morphological analysis).The 20× objective was used for overall microglia soma counting, size quantification, blood vessel association and BBB assessment. Additional 8 FOVs per animal were acquired in the frontal sections for the BBB breakdown assessment covering the medial prefrontal and motor cortex. High-resolution images were acquired with the 40×/oil objective, thereby 30–40 microglial cells were analyzed. Image analysis of VAMs were performed using open-source ImageJ software and custom Python3-based routines. For quantifying microglial soma size (IBA1^+^ cells), we applied automatic thresholding and size filtering > 60 µm^2^. Since IgG levels highly varied between individuals, we rather applied a fixed threshold (threshold = 25) and size filtering > 5 µm2 for BBB breakdown assessment. Vessel segmentation in STL-labelled images was performed using the OMELETTE framework [40] (https://gitlab.com/hmattern/omelette). To adapt the filter's vessel sensitivity parameter gamma for robust vessel enhancement across varying pixel intensity distributions, gamma was empirically set to 40% of the image's maximum absolute Hessian eigenvalues. A multi-scale Frangi filter [41] was then applied with filter scales of 1, 2, 3, 4, and 5 pixels. Blood vessels were subsequently segmented from the enhanced images using hysteresis thresholding [42]. This thresholding employed the three-class Otsu's method [43] to estimate the lower and upper thresholds from the enhancement distribution (self-tuned threshold estimation). To analyze the three-dimensional (3D) microglia branching morphology in rodents, we applied the 3DMorph automatic analysis workflow via MATLAB [44]. All analyses were conducted in a blinded manner with respect to pathological groups.

### Histology and neuropathological evaluation in human post-mortem tissue

Routine neuropathological investigations were performed using hematoxylin & acid fuchsine (modified H&E), advanced silver stains, and single- and double-label immunohistochemistry as previously described [45, 46]. Briefly, brains were fixed in a 4% formaldehyde solution and cut into approximately 1 cm-thick coronal slabs. Tissue slabs and blocks of the frontal, mid-hemispheric, and occipital regions, the cerebellum and various brainstem regions (rostral medulla, pontine-mesencephalic junction, and midbrain) were embedded in polyethylene glycol (PEG 1000, Merck, Carl Roth Ltd, Karlsruhe, Germany). Multiple 100 µm thick consecutive sections were obtained from each embedded tissue block with a sliding microtome (Jung, Heidelberg, Germany). Enlargement of subcortical perivascular spaces, hyalinosis of subcortical perforating vessels in the white matter and basal ganglia area as well as presence of WML were assessed in the modified H&E stain [45]. Stages of Alzheimer-related neurofibrillary changes were visualized using the Gallyas silver stain [33]. Extracellular deposits of Aβ peptide were immunostained with the mouse anti-β-amyloid 17–24 antibody (1:5000, clone 4G8, BioLegend, Koblenz, Germany). Alpha-synuclein pathology was detected with the anti-syn-1 antibody (1:2000, clone 42; BD Biosciences, CA, USA). To visualize microglia and vessels, sections were treated with 10% methanol and 3% concentrated H_2_O_2_ in Tris-buffered saline (TBS). Epitopes were unmasked using pretreatment with 1.3 µg/ml proteinase K for 10–15 min at 37 °C (Invitrogen, Darmstadt, Germany). After blocking of unspecific binding sites with bovine serum albumin (BSA), sections were incubated with the primary antibody against IBA1 (1:500, Abcam, Cambridge, UK) over night, a secondary biotinylated antibody (1:200; 2 h, room temperature, Vector Laboratories, Burlingame, CA, USA), and the avidin–biotin-peroxidase complex (ABC Vectastain, Vector Laboratories, Burlingame, CA, USA). The immunoreaction was visualized using 3,3′-diaminobenzidine tetrahydrochloride (DAB; Sigma Taufkirchen, Germany). Next, sections were washed with TBS at 95 °C for 5 min, retreated with 10% methanol and 3% concentrated H_2_O_2_, and incubated for 48 h with Ulex europaeus lectin I (UEA-l; 1: 800, biotin-coupled, GeneTex, Irvine, CA, USA). Subsequently, sections were incubated with the ABC kit solution, and the reaction product was visualized with a blue chromogen (SK-4700, Vector Laboratories). Omission of all primary antibodies and the lectin resulted in lack of staining.

### Image acquisition in human post-mortem tissue

Images were taken using an Eclipse LV100ND microscope equipped with a digital DS-Fi3 camera and the NIS-Elements software (NIKON GmbH, Düsseldorf, Germany) from the cingulate gyrus of 100 µm-thick hemisphere sections labeled with double-label immunohistochemistry for IBA-1 and UEA-l. The position for image acquisition was randomly selected with the 4× objective and z-stack images were acquired with the 20× objective (image area size 675,470 µm^2^). The N.I.H. ImageJ software was used to generate minimum intensity projections (MIP) from the z-stack images after image brightness was optimized, noise reduction was performed (despeckle command), and the median filter (radius 2.0) was applied in z-stacks. Next, Adobe Photoshop was used for autothresholding and autocontrasting, and to invert the colors of MIP images. The latter step allowed obtaining microglia and vessels with bright colors in a dark background to quantify microglia morphologies with established image analysis pipelines. Using ImageJ, the background of inverted images was subtracted (rolling ball radius 50.0 pixels, using „separate colors “ and „sliding paraboloid “ options), and images were reopened in Adobe Photoshop. The inverted blue color of the microglia was assigned green, and the inverted magenta color of the vessels was assigned red using the channel mixer in Adobe Photoshop to further improve the color contrast between microglia and vessels. Inverted images with double labeling were used to quantify the overall microglia cell density and the density of VAMs (analyzed in MIP generated from z-stacks with 25 images; 2880 × 2048 pixels with a voxel size of 0.11 × 0.11 × 1.5 µm^3^). The VAMs were defined as IBA1-positive cells with their somata attached to UEA-l-labeled vessels. To analyze single-cell microglia morphologies, new inverted images were generated (19–53 images in z-stack) to maximize the number of microglia analyzed per case in the MIP images (larger stacks in cases with lower microglia densities and vice versa to avoid overcrowding/overlay of cells). From these latter images with IBA1-positive cells (green) and UEA-l-labeled vessels (red), red vessels and shades (out-of-focus areas in MIPs) were removed by using the “remove color” function with thresholding in Adobe Photoshop. To characterize microglia morphology, the somata were manually outlined for each cell and quantified using the measure function. To investigate the branching complexity of individual cells, microglia were automatically thresholded using the Triangle option and size filtering of > 2000 pixels, so that the single-cell branching area as well as the cellular solidity, i.e., cellular branching area/convex hull area, could be estimated. For simplicity and consistency, the cellular solidity is called the two-dimensional (2D) ramification index in the rest of this article since it corresponds to the 3D ramification index in rodents. The analyses were performed blinded to the pathological groups.

### Hierarchical agglomerative clustering and uniform manifold approximation and projection (UMAP) for morphological features

We standardized single-cell morphology features and conducted hierarchical agglomerative clustering with Ward-linkage to explore morphological microglia clusters. A pipeline for dimensionality reduction was implemented in Phyton 3.9 [47]. UMAP analysis was performed for both the hippocampal CA1 region and retrosplenial cortex individually. The clustering performance was assessed by generating scatter plots in the UMAP-based latent space with color-coded clusters for visual representation.

### Tissue collection and brain single-cell isolation for FACS analysis

The experimental cohort consisted of 22 rodents, including 5 SHRSP rats in the early hypertension group, 6 SHRSP rats in the late hypertension group and their corresponding age-matched normotensive controls. Each stage was performed twice. Animals were anesthetized with pentobarbital (40 mg/kg) and perfused with sterile PBS. Brains were divided into two sagittal halves: one for flow cytometric analysis, the other for microvessel isolation. Meninges were removed from the hemisphere for flow cytometry and dissected into cortex and hippocampal regions. Each region was collected in separate tubes. Tissues were processed as previously described [48]. Prior to fluorochrome-conjugated antibody labeling, cells were incubated for 15 min at 4 °C with a purified mouse anti-rat CD32 FcγII (clone D34-485, Rat BD Fc Block) to block unspecific binding. Thereafter, cells were stained with a mixture of fluorochrome-conjugated antibodies in 100 μl of FACS buffer for 30 min at 4 °C. The mixture of antibodies included Zombie NIR™, anti-CD45 (clone: OX-1), anti-CD11b (clone: OX-42), anti-CX3CR1 (clone: SA011F11), anti-P2Y12R (polyclonal), anti-CD86 (clone: 24F), anti-CD200R (clone: OX-102), anti-RT1B (clone: OX-6), and anti-CD163 (clone: ED2). Single live cells were identified by the exclusion of doublets, cell debris, and gating of viable cells using a live/dead dye (Additional file 5: Fig. S4). Traditionally, microglia are identified as CD11b^+^CD45^int^, whereas CD11b^+^CD45^high^ population corresponds to other central nervous system (CNS) macrophages [49]. However, microglia respond to inflammatory states upregulating CD45 [50, 51], resulting in incorrect classification of microglia as bone marrow-derived macrophages. Therefore, microglia were identified based on FSC and CD45 expression, with P2Y12R used as a specific marker for the hippocampus (Fig. 3a, b) and cortex (Fig. 3e, f). Quality control included antibody titration and FMO controls. Data were acquired and analyzed using AttuneNxT and Flowjo Analysis Software (v10.5.3).

### Population identification and high-dimensional data analysis

Sample data were acquired, compensated, and subjected to unbiased analysis as previously described [52, 53]. Manually annotated gatings were used to determine microglia and leukocyte frequencies. Live single microglia cells were exported for dimensionality reduction. UMAP visualization was performed for both hippocampus and cortex regions separately. Subsamples of 7,500 and 10,000 microglia cells per animal were used for the hippocampus and cortex, respectively.

### Tissue dissociation for microvascular cell isolation

The brain was divided into two sagittal halves, with one hemisphere processed for FACS as described earlier, and the other dedicated to microvessel isolation [54], following a modified protocol for enhanced purity. Briefly, cerebral cortices were meticulously prepared, removing meninges, cerebellum, and brain stem, then mechanically reduced into ∼1 × 1 mm pieces. These tissue fragments were subjected to digestion in DMEM/F12 containing collagenase type II (1 mg/ml), DNase I (15 μg/ml), penicillin (100 units/ml), streptomycin (100 μg/ml) and glutamine (2 mM), while being mechanically dissociated for 50 min at 37 °C. Subsequently, myelin and neurons were separated by centrifugation in 20% BSA-DMEM/F12 at 1000 g for 20 min at 4 °C. To optimize microvessel retrieval, the myelin layer and BSA supernatant underwent two additional centrifugation cycles under the same conditions. After the third centrifugation, microvessels were combined into a new sterile tube and further digested with collagenase-dispase (1 mg/ml) and DNase I (6.7 μg/ml) in DMEM for 60 min at 37 °C. Meanwhile, a 33% continuous isotonic Percoll density solution was prepared (30,000 g, 60 min, 4 °C), and the digested homogenate was layered on top of the 33% gradient and centrifuged for 10 min at 1000 g at 4 °C with slow deceleration. Microvessels were collected, washed twice in ice-cold DMEM, and filtered through a 40 μm cell strainer for microvessels collection as previously described [55]. The cell strainer was reversed and washed into a new tube to retrieve microvessels. Following a quick evaluation under a bright field microscope to confirm purity, the microvessels were resuspended in 200 μl of RNA*later* (AM7020, Thermo Fisher) for subsequent RNA isolation.

### RNA isolation from cortical microvessels

Microvessels were pelleted at 20,000 g for 10 min and resuspended in 350 μl of RLT Plus Buffer from the RNeasy^®^ Micro Kit (QIAGEN Inc.). Total RNA was isolated using the RNeasy^®^ Micro Kit according to the manufacturer’s instructions. Samples were dissolved in an appropriate amount of RNAase-free ddH_2_O, and RNA concentration and purity were determined using NanoDrop 2000 spectrophotometer (Thermo Fisher) and stored at − 80 °C until further use.

### RT-qPCR

Gene expression levels of tight junction proteins and adhesion molecules were conducted in triplicates using 10 ng isolated RNA. Relative gene expression was determined using the TaqMan^®^ RNA-to-CT™ 1-Step Kit (Thermo Fisher Scientific) using a LightCycler^®^ 96 (Roche). Reverse transcription was performed for 30 min at 48 °C, followed by inactivation for 10 min at 95 °C. Subsequently, a two-step amplification was run for 55 cycles, comprising of denaturation for 15 s at 95 °C and annealing/elongation for 1 min at 60 °C. TaqMan^®^ Gene Expression Assays used for mRNA amplification are listed in Additional file 1: Table S3. For quantification analysis, the threshold cycle (Ct) using the comparative 2^−∆∆Ct^ method was used [56]. Relative target gene mRNA levels were determined by calculating the target gene/reference gene ratio. The resulting data were then normalized to appropriate control groups and presented as fold changes in arbitrary values.

### Statistical analysis

All data points were assessed for Gaussian distribution using the Shapiro–Wilk test. For two group comparisons, the statistical significance was analyzed using a two-tailed Student’s *t* test with Welch’s correction for unequal variances. For conducting multiple comparisons in rodents, a two-way analysis of variance (ANOVA) was performed, followed by Holm-Sidák’s post hoc test. Group and age were considered as categorical variables to examine the statistical significance between hypertensive vs. control rats and to identify potential age-related effects. Data analysis was performed using GraphPad Prism software v.9.3.1 (San Diego, CA). The rodent data shown are representative of four independent experiments, two per time point. All quantified values are represented as independent data points and as mean ± SEM unless specified otherwise. *P* values were considered to indicate statistical significance: * for *p* ≤ 0.05; ** for *p* ≤ 0.01; *** for *p* ≤ 0.001; **** for *p* ≤ 0.0001.

### Software

Flowjo UMAP and Phenograph plugins. Flowjo v.10. Installation of R and R libraries: flowCore, FlowSom, pheatmap, matrixStat. Phyton 3.9. Open-source UMAP package via Phyton (https://umap-learn.readthedocs.io/en/latest/index.html). MATLAB-based Cyt3 software. Open-source script 3DMorph via MATLAB. ImageJ: Rasband, W.S., (https://imagej.nih.gov/ij/, 1997–2018).

## Results

### Distinct microglial morphological features in the cortex of patients with cerebral small vessel disease

**Fig. 1:**
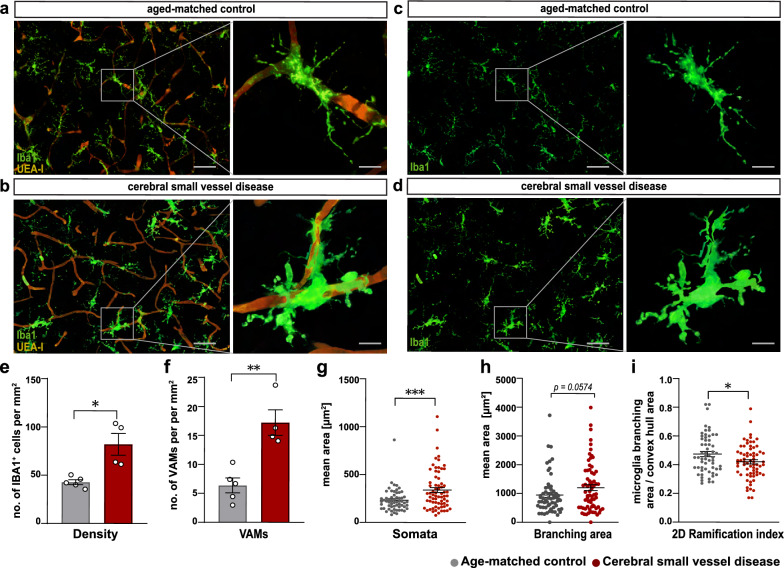
Distinct morphological features of microglia, including enlarged somata thickened processes, and increased territorial volume, observed in post-mortem tissue from patients with cerebral small vessel disease. (**a**) Representative overview and micrographs of individual vessel associated microglia (VAMs) in post-mortem tissue of aged controls and (**b**) cSVD individuals. (**c**) Representative micrographs of individual IBA1^+^ microglia of aged controls and (**d**) cSVD individuals. (**e**) Quantification of microglia density within a defined area of 1,013,205 µm^3^ standardized to 1 mm^2^. (**f**) Quantification of VAMs within the defined region. (**g**–**i**) Quantification of different morphological features of microglia (soma size, average branch area and ramification index). Individual data points indicate averaged microglia data from an individual donor (**e**, **f**) or individual microglia within the defined region (**g**–**i**). Columns and error bars show mean ± SEM; (**a**–**f**), n = 5 controls, n = 4 cSVD subjects. (**g**–**i**), n = 145 microglia in age-matched controls, n = 222 microglia in cSVD. *p*-values: * ≤ 0.05; ** for *p* ≤ 0.01; *** for *p* ≤ 0.001; (scale bar overview image = 100 µm; single cell = 25 µm)

We conducted an in-depth investigation into the potential alterations of microglia density and morphological complexity using IBA1^+^ cells from the cingulate cortex of elderly individuals diagnosed with cSVD (Fig. 1), which showed white matter lesion (WML) as well as hyalinosis of vessels and enlarged perivascular spaces in the subcortical white matter and basal ganglia (Additional file 3: Fig. S1). Our analysis revealed a significant increase in microglia density in cSVD tissue samples (Fig. 1a–e). In addition, we quantified the total number of VAMs (Fig. 1g–i) and determined their density with respect to the total number of IBA1^+^ cells quantified in a defined area, and found a significant increase of VAMs in cSVD individuals compared to control cases without cSVD (Fig. 1f). Furthermore, morphological analysis of individual microglial cells (Fig. 1c, d) revealed that IBA1^+^ cells from cSVD cases exhibited a significantly enlarged somatic area compared to controls (Fig. 1g) and displayed a decreased 2D ramification index (Fig. 1h, i).

### Morphological changes in hippocampal and cortical microglia are already visible in early hypertension in the SHRSP model of cSVD

**Fig. 2:**
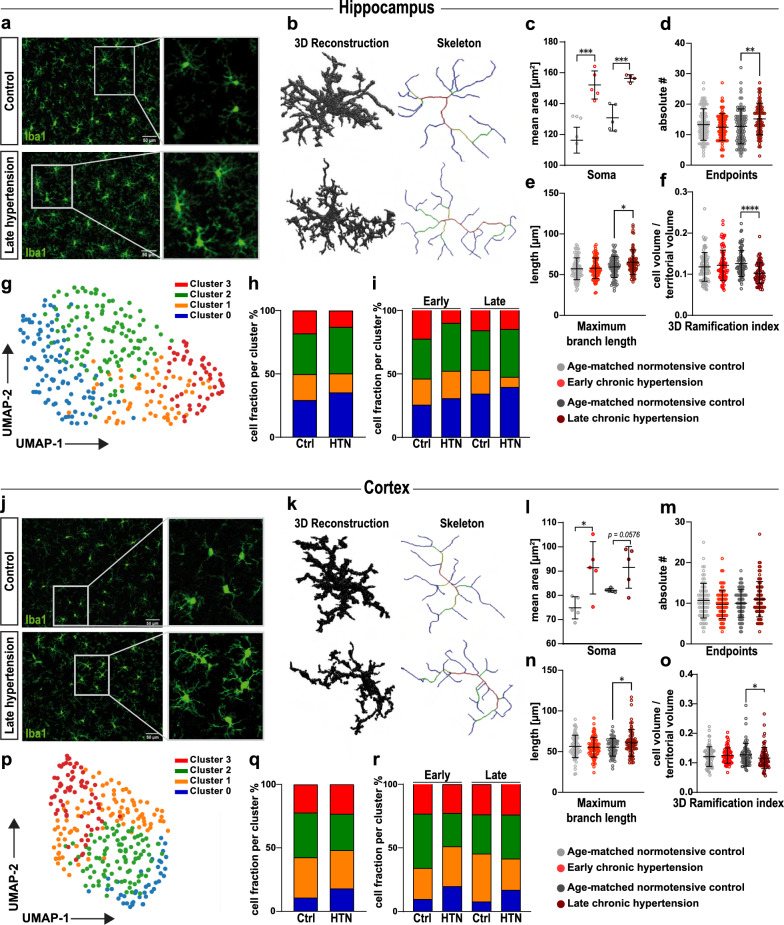
Microglia morphological responses in hippocampal and cortical subfields in chronic hypertensive SHRSP rats. Representative high-resolution images of 15–20 microglia cells of 4 fields of view per animal in the hippocampus (**a**) and in the cortex (**j**). Confocal three-dimensional reconstructions of IBA1^+^ microglia (green) in late chronic hypertension showing altered skeletal geometrical features and morphological changes induced by chronic hypertension in the hippocampus (**b**) and in the cortex (**k**). (**c**–**f**) Quantitative microglial morphological changes derived from 3D microglial reconstructions in the hippocampus and in (**l-o**) the cortex. 15–20 microglial cells were analyzed from 5 independent rodents for each group and brain region. Individual data points indicate averaged microglia data from an individual donor (**c** and **l**; soma area) or individual microglia within a defined region (**d**–**f** and **m**–**o**; endpoints, max branch length, and ramification index). (**g**) UMAP plot of microglia clusters based on major morphological features in the hippocampus and (**p**) the cortex. (h) Relative frequencies of microglia per cluster in the hippocampus and (**q**) the cortex. (**i**) Microglia morphological cluster distribution according to the phase of hypertension in the hippocampus and (**r**) the cortex. Data represent mean ± SEM. Ctrl, Controls; HTN, Hypertension. *p*-values: * ≤ 0.05; **for *p* ≤ 0.01; *** for *p* ≤ 0.001; **** for *p* ≤ 0.0001

Animals in the SHRSP group exhibited elevated systolic blood pressure from 8 weeks onwards compared to age-matched controls (8- and 24-weeks *p* < 0.001, 34-weeks *p* = 0.001, Additional file 3: Fig. S2). We performed single-cell morphological analyses using high-resolution confocal images to investigate the effect of chronic hypertension on microglial morphology and distribution in the hippocampal CA1 region and retrosplenial cortex (Fig. 2 and Additional file 4: Fig. S3). Using unsupervised clustering of all available single-cell morphological features, we obtained four different clusters. These clusters reflect a continuous morphological transition instead of strictly distinct morphological categories (Fig. 2g–i, p–r). Assessing morphological features separately, we observed a significantly increased microglial somatic area, a cell activation marker, in both brain regions already in early chronic hypertension, which persisted in late chronic stages. Additionally, microglia in late chronic hypertension displayed a significantly more ramified phenotype compared to controls in both brain regions (increased number of endpoints/maximum branch length and decreased 3D ramification index) (Fig. 2c–f, l–o). These results are in line with the morphological alterations found in human analyses and suggest that chronic hypertension induces microglial morphological changes.

### Phenotypical characterization of microglia in chronic hypertensive states

**Fig. 3:**
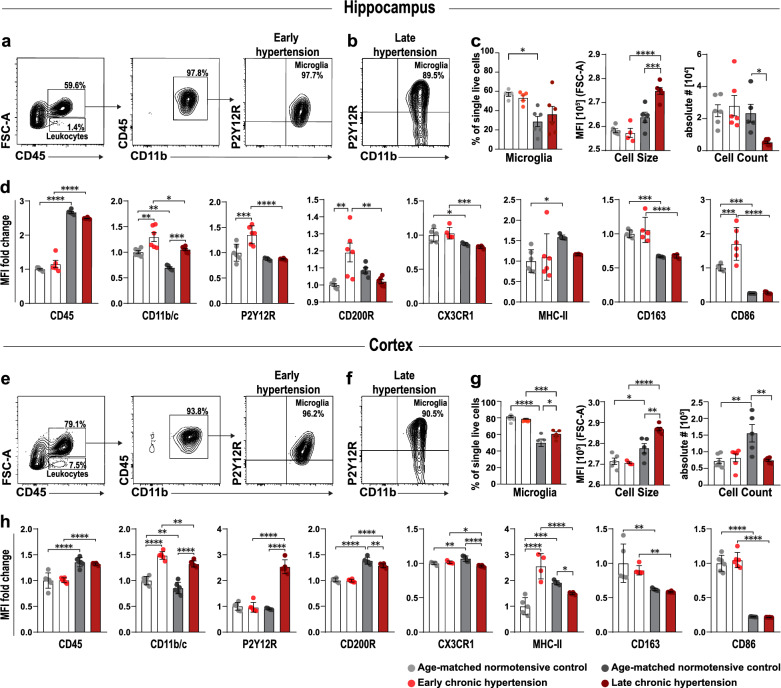
Hippocampal and cortical microglia cell reactivity, age-related effects and dynamic activation in chronic hypertensive states. (**a**, **e**) Identification of microglia derived from the hippocampus and cortex of SHRSP rats via FACS analysis. Single live cells were identified by the exclusion of doublets, cell debris and gating of viable cells (Additional file 4: Fig. 3)*.* Main populations of cells were identified through Forward Scatter light (FSC) and CD45. Leukocytes were selected based on their size and CD45 expression. Higher FSC CD45^+^ events were further gated as CD45^+^ and CD11b/c^+^ positive events and further classified by their expression of P2Y12R and CD11b/c. Double positive events were classified as microglia in early chronic hypertension and (**b**, **f**) late chronic hypertension. (**c**) Frequency and count of microglia cells in the hippocampus and (**g**) the cortex, median fluorescence intensity (MFI) for FSC was used to investigate microglia size. (**d**, **h**) Bar charts showing the fold change in MFI in each surface antigen investigated in hippocampal and cortical microglia. Individual values shown in the graph represent mean ± SEM. *p*-values: * ≤ 0.05; ** for *p* ≤ 0.01; *** for *p* ≤ 0.001; **** for *p* ≤ 0.0001

Next, we characterized microglial populations by analyzing their surface marker expression in both early and late chronic hypertensive states by flow cytometry (Fig. 3a, b, and e, f). Chronic hypertensive states revealed intriguing regional differences at an early stage of hypertension, whereas similarities between the hippocampus and cortex were observed in the control groups. Median fluorescence intensity (MFI) for FSC was used to investigate microglial physical properties. In the hippocampus, aging led to a reduction in microglia frequency. Despite the reduction in microglia counts (% of single live cells) observed in the hypertensive hippocampus during late stages of hypertension, a notable enlargement in microglial cell soma size was observed as the duration of hypertension persisted, and this size difference was significant when compared to normotensive hippocampus (Fig. 3c). In the cortex, microglia frequency decreased during aging in both normotensive and hypertensive brains, but chronic hypertension was associated with a higher microglia frequency (Fig. 3g). Moreover, comparable to the findings in the human cSVD cortex (Fig. 1g), chronic hypertension resulted in an increase in microglial cell size in both the hippocampus and cortex. Interestingly, aging led to a decrease in microglia frequency but an increase in cell size and count in the brains of aged control rats when compared to their young counterparts (Fig. 3c, g). Subsequently, we focused our analysis solely on CD45−, CD11b/c−, and P2Y12R-gated microglia to investigate the density of markers associated with anti-inflammatory and pro-inflammatory microglial pathways.

### Age-dependent and hypertension-dependent regional differences in microglial surface marker expression profile

To characterize microglia in homeostasis compared to early and chronic hypertension, we compared the expression of distinctive markers that indicate dynamic stages based on the median expression of eight proteins (CD45, CD11b/c, P2Y12R, CX3CR1, CD200R, CD163, MHCII and CD86) (Fig. 3d, h). Both hippocampal and cortical microglia undergo an age-dependent increase in CD45 expression, and both hypertensive and normotensive controls exhibit age-dependent decreases in CD11b/c, CX3CR1, CD163, and CD86 surface markers. In early hypertension, hippocampal microglia had increased expression of P2Y12R, which was later suppressed, while cortical microglia did not show any significant difference in P2Y12R expression at this stage. CD200R and CD86 were upregulated in early chronic hypertension in hippocampal microglia, whereas cortical microglia showed no significant difference in these markers. The expression of MHC class II molecules in the CNS was age-dependent in both the hippocampus and the cortex. These differences indicate that hippocampal microglia react earlier and more robustly to chronic hypertension than cortical microglia.

### Phenotypic variations in microglia clusters in the hippocampus

**Fig. 4:**
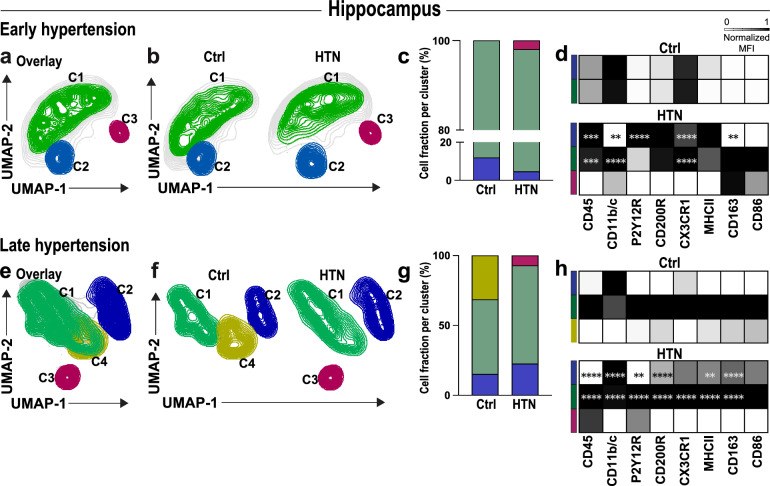
Chronic hypertension results in microglia dynamical changes in the hippocampus of hypertensive rats. Overlay of Uniform manifold approximation and projection (UMAP) map displaying randomly sub-sampled microglia cells from normotensive and hypertensive hippocampus analyzed by flow cytometry in (**a**) early and (**e**) late hypertension. (**b**, **f**) UMAP plot with color-coded Phenograph-guided clustering with cell identities established based on the investigated surface markers. Representative UMAP plots from normotensive and hypertensive brains displaying microglia sub-clusters corresponding to each cohort. (**c**, **g**) Relative frequencies of microglial cells and their respective percentages per cluster in early and late chronic hypertension. (**d**, **h**) Heat map displaying normalized median expression values for each population present in each color-coded cluster exhibiting dynamic expression of diverse microglia activation markers (CD200R, CD45, CD86, CD163, MHCII, CD11b/c, P2Y12R, CX3CR1) upon early and late chronic hypertension compared to their respective age-matched normotensive control. Ctrl, Controls; HTN, Hypertension. *p*-values: ** for *p* ≤ 0.01; *** for *p* ≤ 0.001; **** for *p* ≤ 0.0001

After traditional manual gating analysis of microglia, we applied an additional unbiased multidimensional approach to interrogate the composition and phenotypic variations of microglia subpopulations in the different brain regions. Microglia subpopulations were visualized by UMAP in early (Fig. 4a–d) and late chronic hypertension (Fig. 4e–h). This computational approach yields cells of similar phenotypes to be localized into similar coordinates, facilitating the qualitative assessment of different sub-populations inside the microglia population after automated clustering using Phenograph. Discrete phenotypic variations were identified within the microglia sub-populations, and the co-expression of surface molecules measured on each sub-cluster was plotted into a heatmap for detailed sub-cluster visualization (Fig. 4d, h; Additional file 6: Fig. 5a). Our results revealed the existence of a distinct group of microglial cells expressing specific surface markers (CD86, CD11b/c, and CD163) only in early hypertension, represented by cluster 3 (C3) (Fig. 4b, c). A reduction in the population density of microglial cells within cluster 2 (C2) was also detected during the early stage of hypertension, possibly due to a significant proportion of these cells undergoing transition, as indicated by their allocation to cluster 3 (C3). Further analysis revealed that cluster 2 (C2) exhibited an increased expression of P2Y12R and CX3CR1 compared to normotensive microglia (Fig. 4), suggesting that the upregulation of P2Y12R observed in the overall hippocampal microglial assessment (Fig. 3d) was derived from this specific cluster.

**Fig. 5:**
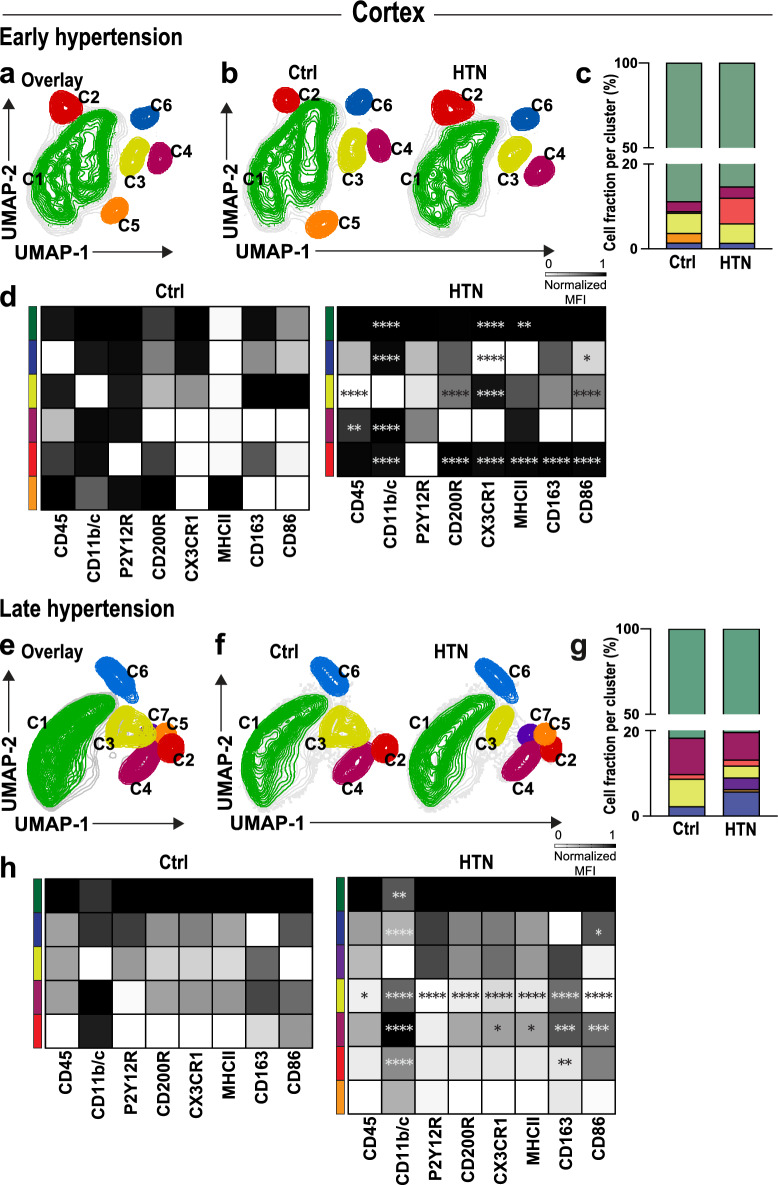
Chronic hypertension results in microglia dynamical changes in the cortex of hypertensive rats. Overlay of Uniform manifold approximation and projection (UMAP) map displaying randomly sub-sampled microglia cells from normotensive and hypertensive cortex microglia analyzed by flow cytometry in (**a**) early and (**e**) late hypertension. (**b**, **f**) Representative UMAP plots from normotensive and hypertensive brains displaying microglia sub-clusters corresponding to each cohort. (**c**, **g**) Relative frequencies of microglia cells and their respective percentages per cluster in early and late chronic hypertension. (**d**, **h**) Heat map displaying normalized median expression values for each population present in each color-coded cluster exhibiting dynamic expression of diverse microglia activation markers (CD200R, CD45, CD86, CD163, MHCII, CD11b/c, P2Y12R, CX3CR1) upon early and late chronic hypertension compared to their respective age-matched normotensive control. Ctrl, Controls; HTN, Hypertension. *p*-values: * ≤ 0.05; ** for *p* ≤ 0.01; *** for *p* ≤ 0.001; **** for *p* ≤ 0.0001

Aged normotensive controls showed a subpopulation of microglia absent in late hypertension, cluster 4 (C4), which comprised one-third of the total microglia population. Cells with similar properties cluster together in a 2D UMAP plot, suggesting that this subpopulation may be transitioning from cluster 1 (C1) (homeostatic markers) to cluster 2 (C2) (overall reactivity) (Fig. 4g, h; Additional file 6: Fig. S5b). In late hypertension, a shift in the microglia population from the predominant cluster 1 (C1) to a higher fraction in the cluster 2 (C2) was detected. This shift was accompanied by a decrease in CD200R, CD45, CD163, MHC-II, P2Y12R, and CX3CR1 in cluster 1 (C1) and an increase in CD11b/c expression. Additionally, cluster 2 (C2) showed significantly higher expression of CD200R, CD45, CD163, RT1B (MHC-II), CD11b/c, and P2Y12R, indicating a second reactive microglial cluster. These results show that microglia from cluster 1 (C1) may be transitioning to cluster 2 (C2) with increased reactivity in late hypertension. Furthermore, cluster 3 (C3) expressed a unique combination of CD45, CD86, and P2Y12R, which was not observed in normotensive controls. Overall, our automated clustering approach revealed discrete phenotypic variations and different co-expression patterns of surface molecules at the early and late stages of hypertension. In the hippocampus, the transition from early to late hypertension was characterized by distinct phenotypic variations in microglia cluster 1 (C1), including a shift in the density of microglia predominantly located in cluster 1 (C1) to an increased fraction in cluster 2 (C2) in late hypertension, which indicated the presence of a second reactive microglial subpopulation with heightened reactivity and altered marker expression.

### Phenotypic variations in microglia sub-populations in the cortex

Unsupervised graph-based clustering of cortical microglia cells in early (Fig. 5a–d) and in late chronic hypertension (Fig. 5e–h) created a 2D map of microglia sub-populations within groups. Analysis of microglia during early hypertension revealed six clusters in normotensive brains, while five clusters were observed in hypertensive brains. During late chronic hypertension, five clusters were observed in normotensive brains, while seven clusters were observed in hypertensive brains. These distinct sub-populations of microglia were identified based on their specific expression patterns of pro and anti-inflammatory pathways. Further examination of these sub-populations revealed that the highest fraction of microglia localized similarly between groups in cluster 1 (C1). Notably, some clusters were absent or unique in one group only. For instance, microglia in cluster 5 (C5) (Fig. 5a–c) that expressed CD45, CD11b/c, P2Y12R, CD200R, and RT1B (MHC-II) (Fig. 5d; Additional file 7: Fig. S6a) were entirely absent in hypertensive brains, whereas cluster 2 (C2) was present in both groups, with a greater proportion of microglia localized within this cluster in hypertensive brains. Moreover, cluster 3 (C3) observed in hypertensive brains displayed heightened expression of CD200R and CX3CR1 but lower expression of CD45 and CD86. On the other hand, cluster 4 (C4) seen in hypertensive brains expressed higher levels of CD45 and CD11b/c (Fig. 5d).

Subsequent analysis indicated that during late chronic hypertension (Fig. 5e–h; Additional file 7: Fig. S6b), microglia were again primarily localized in cluster 1 (C1). While a lower fraction of hypertensive microglia was found in cluster 3 (C3), these cells displayed higher reactivity with increased expression of CD200R, CX3CR1, CD163, MHC-II, and CD11b/c (Fig. 5h). Additionally, two clusters of microglia (C5 and C7) were identified, which were absent in normotensive controls. The cluster 7 (C7) expressed high levels of CD200R, CD45, CD163, MHC-II, CD11b/c, P2Y12R, and CX3CR1, while the cluster 5 (C5) expressed CD200R, CD45 and CD11b/c at the same level as cluster 7 (C7), but with lower expression of MHC-II and P2Y12R. The phenotypic variations in microglia clusters observed in the cortex during early and late chronic hypertension exhibited greater complexity compared to the hippocampus, as evidenced by the presence of a larger number of distinct clusters. While both regions displayed unique cluster compositions, the cortex demonstrated a more intricate constellation of sub-populations, with specific clusters absent or unique to hypertensive brains. However, the dynamic changes observed in surface marker patterns during the progression from early to late hypertension demonstrate that the predominant density of microglia in cluster 1 (C1) remained relatively consistent, showing that certain sub-populations arise specifically due to hypertension-induced changes while others are associated to the aging process.

### Microvascular pathology and immune cell infiltration in hypertension-induced BBB disruption

**Fig. 6:**
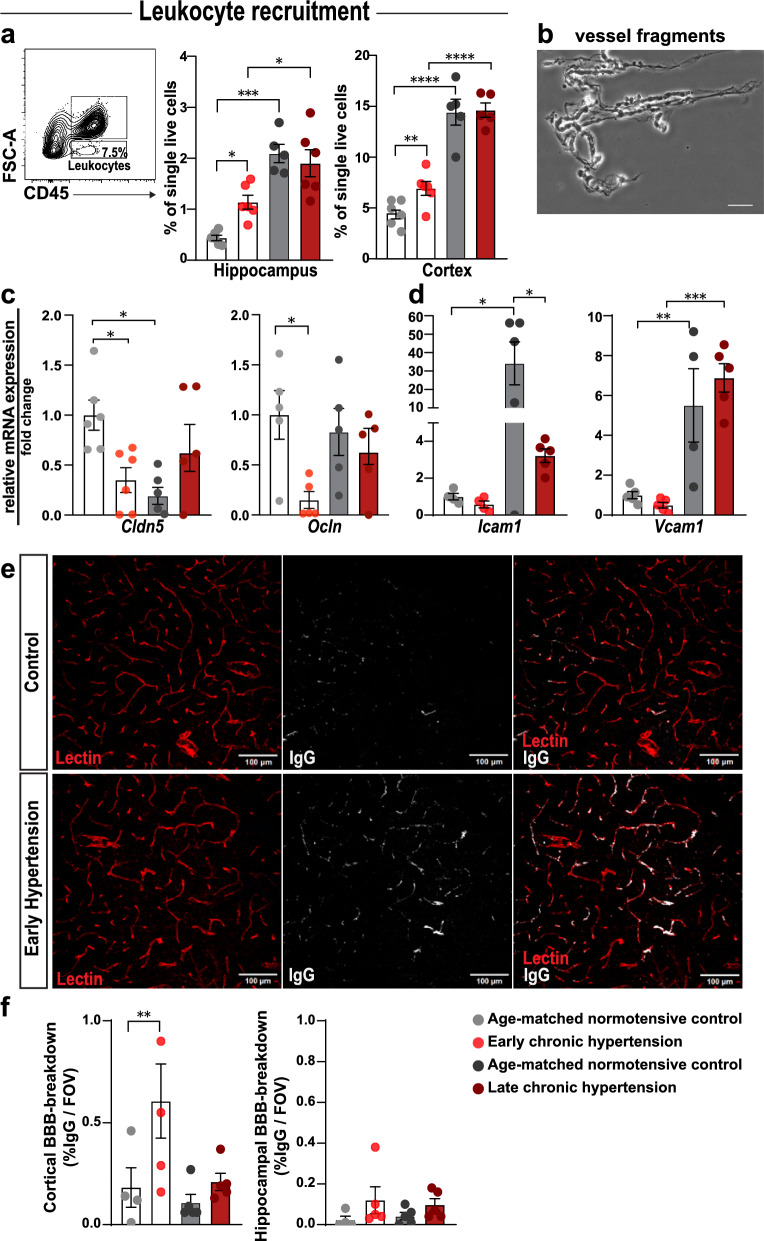
Converging mechanisms of cerebrovascular remodeling and microglia activation in chronic hypertensive states with BBB disruption and leukocyte infiltration into the CNS. (**a**) Identification of leukocyte recruitment into the hippocampus and cortex of all groups in early and late chronic hypertension, represented as the frequency of low FSC CD45.^+^ events from the total single live cells. (**b**) phase-contrast image of isolated microvessel derived from brain cortices plated on a microscope slide (scale bar, 50 µm). Quantification changes in gene expression in cerebral microvessels of tight junction genes (**c**) *Cldn5*, *Ocln,* and adhesion molecules *Icam1* and *Vcam1* (**d**) at early and late chronic hypertension shown by qRT-PCR. In normotensive and hypertensive rat brains. Fold changes were calculated by normalizing gene expression levels to GAPDH. The resulting data were further normalized on mean values of normotensive controls. (**e**) Representative IgG Fluorescence in cortical brain sections (**f**)% of IgG examined per FOV. Bar graphs represent mean ± SEM. Claudin 5; *Ocln*, occludin; *Icam1*, intercellular adhesion molecule-1; *Vcam1*, vascular cell adhesion molecule-1;FOV, field of view; IgG, immunoglobulin G. *p*-values: * ≤ 0.05; ** for *p* ≤ 0.01; *** for *p* ≤ 0.001; **** for *p* ≤ 0.0001

**Fig. 7:**
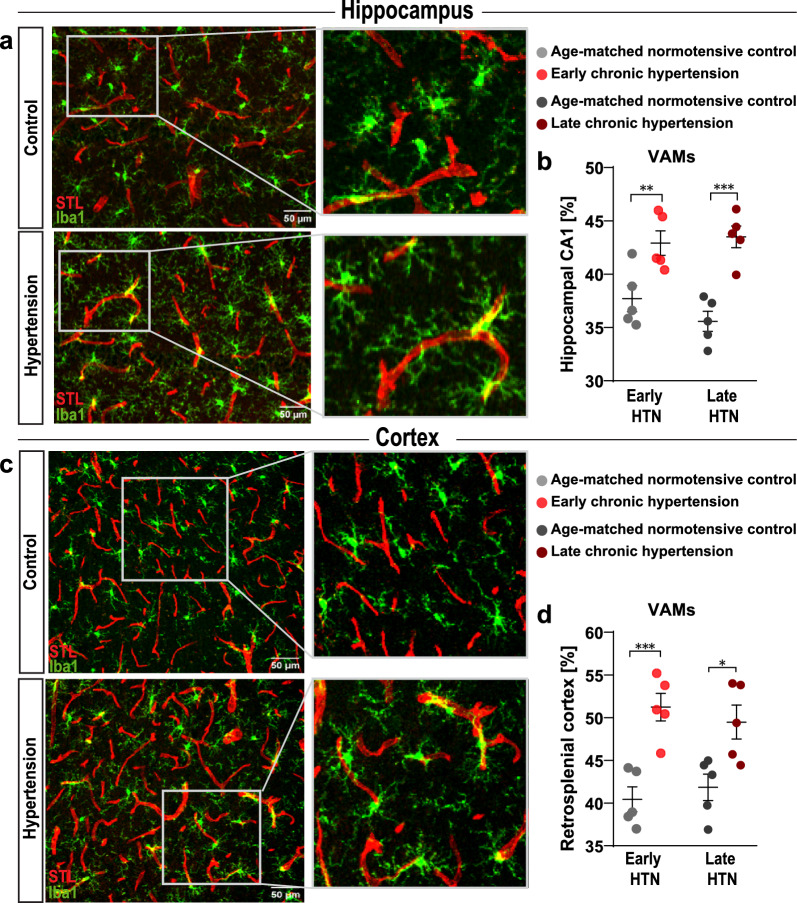
Emergence of vascular-associated microglia in chronic hypertensive states (**a**) Immunofluorescence confocal microscope image of microglia stained with anti-IBA1 representative from hippocampal CA1 region of late hypertensive rats (lower) and its age-matched normotensive control (upper). (**b**) Frequency of vascular-associated microglia (VAMs) in the hippocampal CA1 region. (**c**) Immunofluorescence microglia staining representative from retrosplenial cortex with anti-IBA1 of late hypertensive rats (lower) and its age-matched normotensive control (upper) (**d**) Frequency of VAMs in the retrosplenial cortex. HTN, Hypertension; IBA1, ionized calcium-binding adapter molecule 1 (microglial marker); STL, solanum tuberosum lectin-fluorescein isothiocyanate (endothelial marker). *p*-values: * ≤ 0.05; ** for *p* ≤ 0.01; *** for *p* ≤ 0.001; **** for *p* ≤ 0.0001

An increase in the frequency of leukocytes within the cortex and hippocampus during the early phase of hypertension was detected using flow cytometry, suggesting potential BBB leaks of these cells from the bloodstream (Fig. 6a). To investigate temporal changes in BBB integrity and the correlation of BBB disturbances with microglial responses in chronic hypertension, we examined isolated fragments of microvessels and found a significant downregulation of the two critical tight junction molecules claudin-5 (*Cldn5*) and occludin (*Ocln*) in early hypertension (Fig. 6b, c). Vascular cell adhesion molecule-1 (*Vcam1*) and intercellular adhesion molecule-1 (*Icam1*) were upregulated with increasing age (Fig. 6d). We conducted IgG fluorescence assays in cortical and hippocampal brain sections. In cortical regions (mean of mPFC, MtCx, and RSC), our results revealed a significant difference during early chronic hypertension. The comparison between late chronic hypertension with their normotensive controls revealed no significant changes. In the hippocampal region (CA1), no differences in BBB breakdown were observed between early hypertension or those with late chronic hypertension with their normotensive controls. These results provide further evidence of BBB breakdown and display region-specific variations (Fig. 6e, f; Additional file 8: Fig. S7). Additionally, higher frequencies of VAMs were present in both the hippocampus and cortex in early and late chronic hypertension, similarly as in the human analysis, suggesting the potential involvement of VAMs in vascular (dys)function and associated pathologies. Our results demonstrate a biphasic course of hypertension-induced BBB disruption, immune cell infiltration, and VAM reactivity in the brain in the context of chronic hypertension (Fig. 7a–d).

### Correlations between morphological alterations in microglia and their phenotypic marker expression profiles in the aging brain and in chronic hypertensive states

**Fig. 8:**
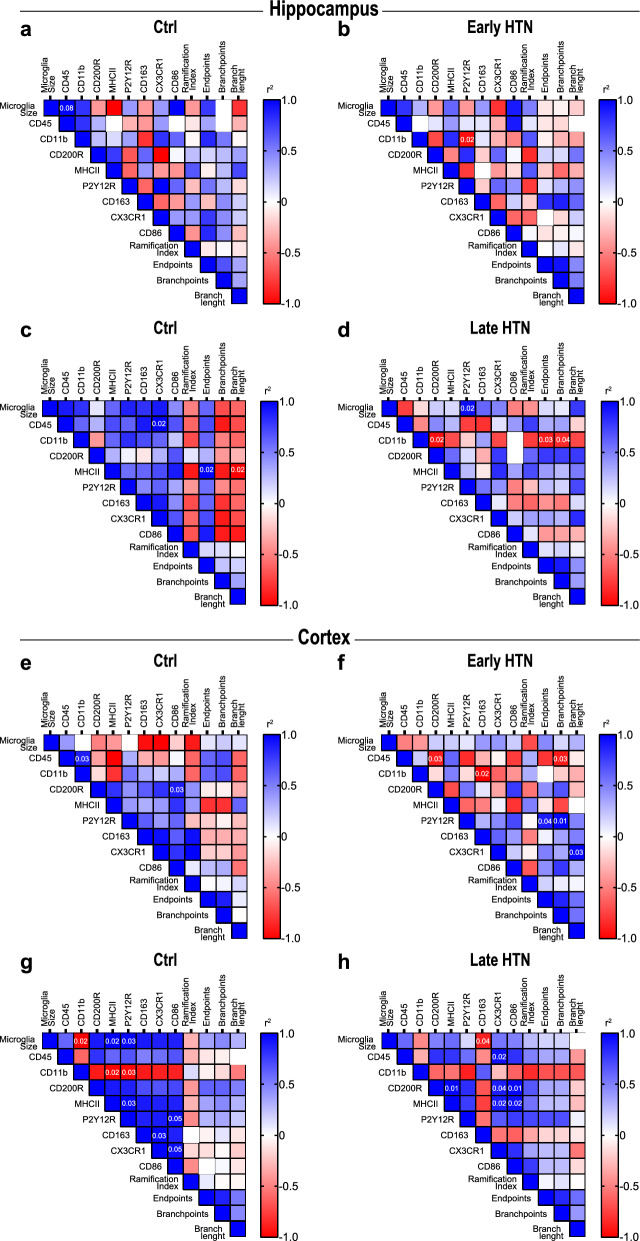
Hippocampal and cortical microglia size and activation markers correlate differently during hypertensive stages. Correlation plots from hippocampal and cortical microglia in (**a**, **e**) young controls, (**b**, **f**) early hypertension, (**c**, **g**) older controls and (**d**, **h**) late hypertension. Statistically significant values (non-parametric Spearman’s test) are shown in their respective cells. The color of each cell represents an estimation of the positive (red) or negative (blue) correlation index based on the r value. Ctrl, Controls; HTN, Hypertension

We assessed the correlation between the expression profile of microglial surface markers and morphological features of microglia in the cortex and hippocampus of normotensive and hypertensive brains (Fig. 8). In the hypertensive hippocampus, early chronic hypertension was associated with positive correlations between CD11b/c and MHC-II expression, suggesting similar microglial activation and antigen presentation. Additionally, a positive correlation between CD200R and P2Y12R expression may indicate a potential neuroprotective role for microglia. In contrast, the negative correlation between CD11b/c and P2Y12R suggested a phenotypic shift from surveillance to a reactive state in the hypertensive hippocampus (Fig. 8b). During late chronic hypertension, the positive correlation between microglial soma size and P2Y12R expression, may reflect microglial hypertrophy and increased chemotaxis, while the negative correlation between CD11b/c and CD200R is suggestive of an interplay between these two markers in regulating microglial activation in the hippocampus (Fig. 8d). In the normotensive aging hippocampus, positive correlations between CD45 and CX3CR1, and between MHC-II and microglial endpoints, may reflect compensatory mechanisms of antigen presentation and dynamics of microglial process, allowing microglia to actively sense and respond to changes in their environment. Moreover, we found a negative correlation between MHC-II expression and microglial branch length, which may further suggest a tradeoff between antigen presentation and process elongation (Fig. 8c). During the early stage of hypertension, we observed negative correlations between CD45 and CD200R and between CD11b/c and CD163 in the cortex, suggesting the involvement of CD45 in downregulation of CD200R signaling pathways and classical activation of microglia. Like in the hippocampus, we found a positive correlation between P2Y12R and microglial endpoints and between P2Y12R and branch points in the cortex that may further indicate the role of this marker in microglial surveillance, phagocytosis, and morphological plasticity (Fig. 8f). At the late stage of hypertension, we observed a negative correlation between microglial soma size and CD163 in the cortex indicative for a shift towards morphological features that facilitates microglial surveillance contributing to tissue repair. The positive correlations observed in the cortex between CD45 and CX3CR1, and between CD200R and MHC-II, CD200R and CX3CR1, and CD200R and CD86 implicated a role in microglial reactivity and interactions with neurons (Fig. 8h). During normotensive aging, we found negative correlations in the cortex between CD11b/c and microglial soma size, and between MHC-II and P2Y12R, which suggested smaller microglia soma area and reduced involvement in antigen presentation and surveillance. In addition, we observed positive correlations between microglial soma size and MHC-II and between microglial soma size and P2Y12R that indicated specialization in antigen presentation and phagocytosis, and the positive correlation between P2Y12R and CD86 implied a role surveillance promoting T-cell activation. Positive correlations between CD163 and CX3CR1 and between CX3CR1 and CD86 suggested microglial involvement in anti-inflammatory responses and tissue repair in the aging cortex (Fig. 8g).

## Discussion

Our study provides an in-depth examination of microglial reactivity during chronic hypertension. It enriches our understanding of microglial dynamics, challenging the one-size-fits-all view of microglial activation. Our research question was primarily focused on characterizing and understanding microglial reactivity in the context of hypertensive cSVD since it had never been done previously. We identified distinct subpopulations based on surface markers characteristic of pro-inflammatory responses, homeostasis maintenance, and repair. Instead of viewing microglia as a continuum of cells responding linearly to certain pathology, we unveil a complex landscape where different microglial subpopulations may exhibit diverse functions. This paradigm shift (recently reported in other diseases [27]) enhances our comprehension of microglial biology, and characterizing the complexity of microglial reactivity can pave the way for novel therapeutic strategies. Additionally, we investigated microglial changes upon aging, which is biologically significant as it aligns with the growing recognition that aging is a critical factor in the development of neurodegenerative diseases. Understanding how microglia adapt during aging and respond to additional challenges like chronic arterial hypertension is essential for comprehending age-related neurological conditions.

In our pursuit to understand the biological implications of microglial reactivity, our study has revealed a link between microglial reactivity and early BBB breakdown. Specifically, we observed the upregulation of CD11c and MHCII in cortex microglia and CD11c and CD86 in hippocampal microglia during the early hypertensive stage. These surface markers, indicative of pro-inflammatory responses, suggest microglial potential involvement in initiating BBB breakdown. The mechanism by which microglia may contribute to local BBB dysfunction resides in the pro-inflammatory state of neighboring microglia, further facilitating the significant transmigration of leukocytes observed in both hippocampal and cortical regions. The presence of these immune cells amplifies the overall inflammatory response, thereby contributing to BBB dysfunction. While causation cannot be definitively established at this stage, the correlation between microglial activation and BBB leaks presents a compelling mechanism for the pathogenesis of vascular cognitive impairment. This observation paves the way for future investigations focused on targeting specific microglial populations to mitigate BBB leakage and neuroinflammation.

### Implications of altered microglial morphological features in cSVD pathogenesis

Our study presents distinct features of microglia in the context of cSVD. Specifically, we detected a higher abundance of microglia with thicker processes and enlarged somata, a decreased ramification index, and a higher association with the vasculature. These alterations in microglial are indicative of a shift in microglial function [20, 57, 58], which may be linked to metabolic changes and their capacity to respond to vascular disturbances. While some studies have reported amoeboid microglia on white matter in response to hypertension [59], our findings in gray matter differ, highlighting potential regional disparities in microglial reactivity. Different rodent models of hypertension consistently showed increased microglial cell volume, accompanied by increased microglia complexity in grey matter [36]. In our rodent model, we consistently observed enlarged microglial somata and increased ramification complexity in gray matter. We confirmed that the enlarged somata and microglial association to the vasculature were morphological features present already at the early stages of hypertension, whereas the increased ramification complexity appeared during the late stage. These changes could impact the glymphatic clearance system, contributing to neurovascular injury. Previously reported findings have indicated that brain tissue surrounding blood vessels in patients with cSVD is affected even before the emergence of lesions such as microbleeds or lacunar strokes [12, 35, 60]. These changes in the extracellular matrix around small brain capillaries can lead to a loss of vascular contractibility, resulting in an inability to meet the high metabolic demands of the aging brain. Evidence indicates that preexisting hypertension exacerbates the development of secondary neurodegeneration beyond its acute effect on neurovascular injury [7, 21]. These findings highlight the importance of how managing vascular risk factors and midlife hypertension is crucial for preserving vascular and brain health homeostasis in older age.

### Revealing the vulnerability of the hippocampus in hypertensive cSVD

Our study reveals the vulnerability of the hippocampus in hypertensive cSVD. Evidence suggests that cognitive decline associated with cSVD often involves memory disturbances and cortical symptoms, such as executive dysfunction and attention deficits [61]. A recent investigation revealed an age-related disruption of the BBB in the hippocampus as an early event in the aging human brain [62]. This early BBB leakage, observed in mild cognitive impairment, may be a relevant starting point to understand similar pathophysiological processes in hypertensive cSVD. Additionally, cardiovascular risk factors impact memory function through WMH [63], potentially enhancing the vulnerability of different brain regions to the combined effect of age and hypertension [64]. Therefore, it is plausible to hypothesize that hypertensive cSVD could exhibit a similar occurrence. Notably, our analysis revealed that even at the early stages of hypertension, microglia displayed a dynamic phenotype. A pro-inflammatory phenotype is evidenced by the upregulation of CD11b/c and CD86, alongside an enhanced chemotactic and anti-inflammatory responsivity as reflected by P2Y12R and CD200R upregulation. These findings exhibit a delicate balance between inflammation and protection in response to early BBB leaks [65, 66].

It is an established finding that the hippocampus is affected in experimental [67, 68] and clinical cSVD [69], carrying direct implications for the development of vascular cognitive impairment. Moreover, blood pressure (BP) management can have a profound impact on cognitive function. For instance, studies have shown that BP lowering through medications like amlodipine can rescue memory impairment associated with hypertension [70]. Notably, consistent with the improvement in memory, amlodipine has been found to reduce microglial activation, suggesting a potential link between microglial reactivity and cognitive function. These findings indicate the need for further exploration into the direct association between hypertension, microglial alterations, and cognitive decline. Our study design, however, could not address the effects of late-life hypertension on the aging brain, which are rather complex [71], and future work is needed.

In line with relevant perspectives [8], our study contributes to the understanding of early changes preceding or following the development of brain arteriolosclerosis (B-ASC) in the context of hypertension. Our data support the interlinkage between hypertension, vessel wall alterations, and neuroinflammation, as recent reviews indicate [8, 10]. Our study extends this view by providing a deeper profiling of the dynamic and region-specific neuroinflammatory responses and microglial phenotypes. By investigating microglial phenotypical changes during the early phases of hypertension, we provide valuable insights into the neuroinflammatory aspects of microvascular disease. Furthermore, our results can aid in the classification of cSVD subtypes and contribute to the development of targeted therapies, highlighting the relevance of identifying early targets to potentially prevent or manage advanced B-ASC stages. This deeper understanding of microglial heterogeneity and dynamics during hypertensive states may have important implications for future therapeutic strategies and personalized medicine in cSVD.

### Age-dependent and pathological changes in cortical microglia marker expression profile suggest microglia reactivity and primed states in chronic hypertension

The interplay between vascular inflammation, arterial function, and age-related diseases is complex and multifaceted [10]. Our findings evidence the age-dependent and pathological changes in cortical microglia phenotype, which indicate microglia reactivity and primed states during chronic hypertension. These changes, particularly the dysfunction in the CD200-CD200R system, are critical for regulating anti-inflammatory signals [72], and are commonly associated with the aging brain. The upregulation of the inhibitory immune receptor CD200R with aging, coupled with its significant reduction in hypertensive brains, implies inefficient regulation of pro-inflammatory conditions that ultimately lead to deficient feedback loops in the induction of inflammatory mechanisms that can contribute to vascular inflammation and neurodegeneration [10, 73–75]. While both the hippocampus and cortex exhibit distinctive microglia cluster compositions, the larger cortex size implies a greater spatial and functional complexity, potentially requiring a more diverse array of microglia subpopulations. Spatial proteomics analysis or multiple-target immunohistochemistry can be a promising approach for future research.

### Microglia heterogeneity in chronic hypertension and its implication for BBB leakage and cerebrovascular remodeling

Microglia, known for their heterogeneity, exhibit significant diversity during pathological conditions [16, 27]. Chronic hypertension, as observed in our study, resulted in a range of phenotypical characteristics. Our findings show that P2Y12R overexpression potentially impacts chemotactic and phagocytic activities. Recent evidence has shown that microglial response to capillary lesions depends on the P2Y12 purinergic receptor (P2Y12R) for BBB repair [76, 77]. Furthermore, P2Y12R accumulation at endothelial cell contacts is essential for vasodilation, and focal loss of P2Y12R has been associated with synaptic degeneration, emphasizing the link between vascular inflammation and synaptic/neural dysfunction [78]. Microglia can exist in various reactive states, including primed and senescent states. Our analysis identified two distinct microglia cell states associated with chronic hypertension, suggesting their contributions to neuroinflammatory signals. Microglia that exhibit pro-inflammatory characteristics are known to undergo morphological changes and secrete cytokines, which can cause significant damage to both the vasculature and neuronal tissue [79, 80]. The resulting reactive microglia phenotype observed in early hypertension may be a cause or a consequence of the significant disturbances seen in the BBB. In one way, microglia expression levels of purinergic receptor P2Y12R observed in chronic hypertensive states may trigger its migration to vascular sites and lead to a “classical” microglia reactivity pattern, modulating its cytoskeletal network for phagocytosis, followed by remodeling of the vessel wall and significant BBB disturbance, with consequent ingress of leukocytes into the CNS perpetuating microglial activation [58]. Conversely, increased leukocyte adhesion and remodeling of the vessel wall due to hypertension-induced systemic inflammation may initiate a microglial “resolving” activation pattern aiding in the resolution of the BBB disturbance that leads to a phase of BBB healing. These findings have implications for BBB leakage and cerebrovascular remodeling in chronic hypertension. Reactive microglia can exacerbate vascular injury, leading to perivascular cell reactivity, ultimately affecting glymphatic clearance and protein deposit removal around the neurovascular unit.

### Concluding remarks

**Fig. 9:**
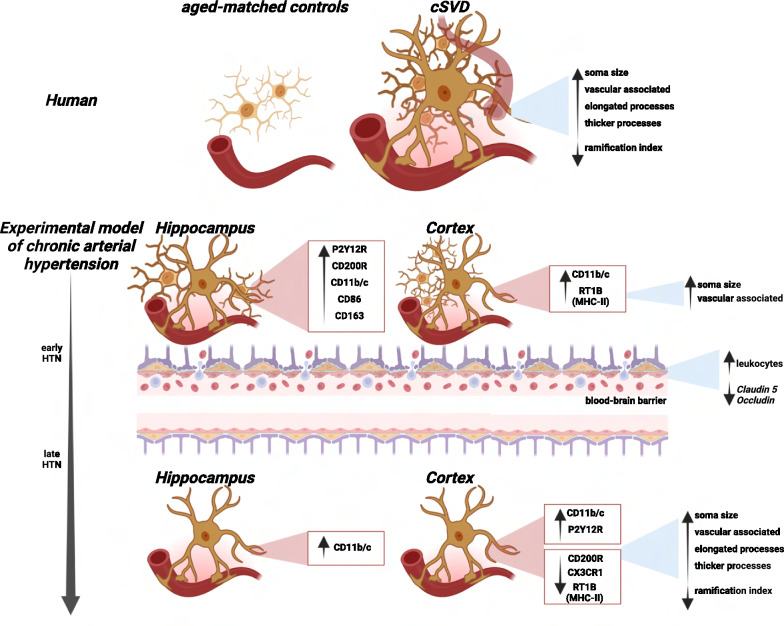
Summarizing figure. Stage-dependent microglia dynamical changes in early and chronic hypertensive states

Collectively, our findings reveal the dynamic nature of microglia in the context of cSVD and highlight the stages of microglial phenotypes in the hippocampus and in the cortex along hypertensive states (Fig. 9). Additionally, these data support the notion that the combination of endothelial alterations during early chronic hypertension, coupled with microglial reactivity and the influence of aging, shapes a synergy that may contribute to the development of cSVD. These insights enhance our understanding of microglial behavior and emphasize the demand for region-specific investigations to discern the complex effects of hypertensive states. Further research is needed to characterize the function of immune cells within the CNS, elucidate vascular cell metabolism during chronic hypertension, and investigate systemic inflammatory markers that could predict the onset and progression of cSVD.

### Strengths and limitations

Extensive analysis of microglia in human post-mortem cSVD tissue was conducted, providing valuable insight into distinctive microglial features in the context of cSVD pathology. By utilizing a murine model, we were able to investigate further and validate the observations made, enhancing the reliability and robustness of the results. This approach strengthens the translational relevance of the study. Furthermore, microglia heterogeneity was investigated by integrating high-resolution morphological variables and single-cell surface protein phenotypic characterization. By employing both supervised and unsupervised analyses, the dynamic changes of the microglia landscape in the hippocampus and cortex of normotensive (Wistar) and chronic hypertensive rats (SHRSP) were examined. To the best of our knowledge, this approach, in the context of chronic arterial hypertension and in rats, fills a significant gap in the current literature. Importantly, these results provide evidence of the diversity within microglia sub-populations that confirms a spectrum of microglia phenotypes associated explicitly with chronic arterial hypertension. This study makes an essential step towards characterizing microglia subsets in normotensive-aged brains as well as in chronic hypertensive states. There are several limitations to be considered in this study. First, the human post-mortem tissue data set had a relatively small sample size, which could limit the generalizability of the findings. However, obtaining a larger sample size in this highly specific population is extremely challenging due to the scarcity of post-mortem tissue from patients diagnosed solely with cSVD without comorbid neurodegeneration. Obtaining human post-mortem brain tissue with confirmed cSVD pathology was challenging, and the availability of such samples was limited. Therefore, while we have made every effort to provide meaningful insights from our human autopsy studies, we aimed to complement and expand them with the animal investigations. Despite the negligible neurodegenerative pathology in the human brain cases included to the study, however, the hippocampus often showed some AD-related changes in the elderly, which is known to cause changes in microglia phenotype, therefore prompting us to limit our investigations to the cortex. Second, while our study successfully identified distinct structural microglial features in chronic hypertensive states, functional assessments were not included in this investigation. The focus was primarily on the phenotypical changes observed in microglia. Further studies should characterize the functional consequences of the identified microglial alterations in the context of cSVD.

### Supplementary Information

**Additional file 1: Table S1**. List of postmortem human patient samples used in the study. **Table S2**. Summary of methodologies employed in rodent brains. **Table S3.** TaqMan assays used for RT-qPCR analyses (DOCX 15 kb)

**Additional file 2: Fig. S1**. Pathological hallmarks of arteriosclerosis (AS) in the human brain visualized in the subcortical white matter with the modified H&E stain in 100µm-thick brain sections. In comparison with control cases (a), overview images show tortuous vessels running in enlarged perivascular spaces (thick black arrow) in the white matter cSVD cases (b). (c) White matter vessel with hyalinosis in the tunica media (thin black arrow). (d) Aggregates of brown hemosiderin in the perivascular space of a white matter vessel indicative of old microbleed (PPTX 4330 kb)

**Additional file 3: Fig. S2**. SHRSP exhibit elevated systolic blood pressure from the age of 8-weeks onwards compared to age-matched Wistar controls. Systolic blood pressure was measured in Wistar rats and SHRSP (n = 3 per group) at 8, 24 and 34 weeks of age by indirect tail-cuff method. Pressure and pulse rate signals were continuously recorded and digitalized using BP-2000 Analysis Software (BP-2000 Analysis System, 4-channels, Visitech Systems, Apex, NC, USA). Systolic blood pressure was determined as the mean of ten cuff inflation measurements. Data are represented as mean ± SEM. Statistical analysis was performed using 2way ANOVA with Holm-Sidák’s post hoc test for group and age comparison. *p*-values: ** for *p* ≤ 0.01; *** for *p* ≤ 0.001 (PDF 139 kb)

**Additional file 4: Fig. S3**. Hippocampal and cortex microglia distribution based on 8 morphological features. (a) Ward hierarchical clustering dendrogram of a total of 600 individual Iba1+ cells representative of hippocampal CA1 region and retrosplenial cortex captured for individual 3D reconstruction and used in further analysis. (b) Relative frequencies of microglia categorized into four distinct morphological clusters in the hippocampus and cortex based on subregions as categorical value. (c) Relative frequencies of microglia in the hippocampus and (d) the cortex at early and late chronic hypertension. Statistical analysis was performed using 2way ANOVA with Holm-Sidák’s post hoc test for region and cluster comparison. *p*-values: * ≤ 0.05; *** for *p* ≤ 0.001 (PDF 194 kb)

**Additional file 5: Fig. S4**. Initial gating strategy and identification of Microglia in normotensive hippocampus and cortex. (a) Representative flow cytometric analysis of isolated cells derived from normotensive and hypertensive brains. Cells were selected according to their size and granularity in the forward (FSC-A) and side light scatters (SSC-A). Thereafter, single cells were selected in regard to the ratio of their cell size vs. cell signal displayed in the FSC-H/FSC-A plot. Finally, dead cells were identified by their high affinity to live/dead dye resulting in a brighter fluorescence than live cells. Only live cells were selected for further analysis. (b, c) Identification of microglia derived from the hippocampus and (d, e) cortex of normotensive controls via FACS analysis (PDF 313 kb)

**Additional file 6: Fig. S5**. Chronic hypertension results in microglia dynamical changes in the hippocampus of hypertensive rats. Bar charts showing the median fluorescence intensity (MFI) of each surface antigen investigated in each hippocampal microglia cluster in early (a) and chronic hypertensive stages (b). Bar charts represent mean ± SEM. Ctrl, Controls; HTN, Hypertension. *p*-values: ** for *p* ≤ 0.01; *** for *p* ≤ 0.001; **** for p ≤ 0.0001 (PDF 146 kb)

**Additional file 7: Fig. S6**. Chronic hypertension results in microglia dynamical changes in the cortex of hypertensive rats. Bar charts showing the median fluorescence intensity (MFI) of each surface antigen investigated in each cortical microglia cluster in early (a) and chronic hypertensive stages (b). Bar charts represent mean ± SEM. Ctrl, Controls; HTN, Hypertension. *p*-values: * ≤ 0.05; ** for *p* ≤ 0.01; *** for *p* ≤ 0.001; **** for *p* ≤ 0.0001 (PDF 150 kb)

**Additional file 8: Fig. S7**. Comparative IgG fluorescence in cortical and hippocampal brain regions in chronic hypertensive states. (a) Representative IgG Fluorescence analyzed in cortical brain sections in late chronic hypertension and in hippocampal CA1 region in early (b) and late (c) chronic hypertensive stages. IgG, immunoglobulin G (PDF 685 kb)

## Data Availability

The raw data that supports the conclusions of this article will be made available by the corresponding authors without undue reservation.
