# Supplementary material for: Spatio-temporal dynamics of microglia phenotype in human and murine cSVD: impact of acute and chronic hypertensive states
Source: Acta Neuropathol Commun. 2023 Dec 19;11:204. doi: 10.1186/s40478-023-01672-0 (PMC10729582; doi:10.1186/s40478-023-01672-0)
Supplement: Supplementary file 1 — Additional file 1: Table S1. List of postmortem human patient samples used in the study. Table S2. Summary of methodologies employed in rodent brains. Table S3. TaqMan assays used for RT-qPCR analyses (DOCX 15 kb) [file 40478_2023_1672_MOESM1_ESM.docx]

**Supplementary information**

**Spatio-temporal dynamics of microglia phenotype in human and murine cSVD: impact of acute and chronic hypertensive states**

Lorena Morton*^1^, Philipp Arndt*^2,3^, Alejandra P. Garza^1^, Solveig Henneicke^2,3^, Hendrik Mattern^3,4,6^, Marilyn Gonzalez^1^, Alexander Dityatev^3,5,6^, Deniz Yilmazer-Hanke^7^, Stefanie Schreiber^+2,3,6,8^, and Ildiko R. Dunay^+1,5,6,8#^

**Suppl. Table 1 – List of postmortem human patient samples used in the study**

| **Randomized Case-Code** | **Age** | **Sex** | **Group** | **cSVD Hallmark** | **NFT Stage (Gallyas)** | **Aß Stage** | **Aß Phase** | **PD (LB)** | **Diagnosis** |
| --- | --- | --- | --- | --- | --- | --- | --- | --- | --- |
| Case1 | 54 | f | Control | n/a | I | B | 3 | 0 | Ovarian cancer |
| Case5 | 65 | m | Control | n/a | I | 0 | 0 | 0 | Myocardial infarction |
| Case4 | 71 | f | Control | n/a | 0 | 0 | 0 | 0 | Left heart failure |
| Case8 | 53 | m | Control | n/a | I | 0 | 0 | 0 | Plasmacytoma, pneumonia |
| Case9 | 74 | f | Control | n/a | II | A | 1 | 0 | Myocardial infarction |
| Case6 | 49 | m | cSVD | WML | I | A | 1 | 1 | Arterial hypertension, cardiac arrhythmia |
| Case 7 | 57 | f | cSVD | WML | 0 | 0 | 0 | 0 | Non-hodgkin lymphoma |
| Case3 | 69 | f | cSVD | WML | I | A | 1 | 0 | Pontine bleeding |
| Case2 | 81 | f | cSVD | WML | I | A | 1 | 0 | Post-ischemic infarct (MCA-l) |

Aß – amyloid beta; subcortical cSVD – cerebral small vessel disease; LB – Lewy bodies; MCA – middle cerebral artery; n/a – not applicable; NFT – neurofibrillary tangles; PD – Parkinson’s disease; WML – white matter lesions

**Suppl. Table 2 – Summary of methodologies employed in rodent brains**

|  | **25 weeks** | | **34 weeks** | |
| --- | --- | --- | --- | --- |
|  | *Wistar* | *SHRSP* | *Wistar* | *SHRSP* |
| **One hemisphere for FACS** | **10** | **10** | **12** | **12** |
| **One hemisphere for microvessel and RNA isolation** |  |  |  |  |
| **Whole brain for imaging** | **5** | **5** | **5** | **5** |
| *total n= 64* | 15 | 15 | 17 | 17 |

SHRSP – spontaneously hypertensive stroke-prone Rat; FACS – flow cytometric analysis; RNA – ribonucleic acid

**Suppl. Table 3 – TaqMan assays used for RT-qPCR analyses**

| **Gene Symbol** | **Gene Name** | **Assay ID** |
| --- | --- | --- |
| *Cldn5* | claudin 5 | Rn01753146_s1 |
| *Gapdh* | glyceraldehyde-3-phosphate dehydrogenase | Rn01775763_g1 |
| *Icam1* | intercellular adhesion molecule 1 | Rn00564227_m1 |
| *Ocln* | occludin | Rn00580064_m1 |
| *Vcam1* | vascular cell adhesion molecule 1 | Rn00563627_m1 |
