# Supplementary figures and images for: Spatio-temporal dynamics of microglia phenotype in human and murine cSVD: impact of acute and chronic hypertensive states
Source: Acta Neuropathol Commun. 2023 Dec 19;11:204. doi: 10.1186/s40478-023-01672-0 (PMC10729582; doi:10.1186/s40478-023-01672-0)

## Slide 1
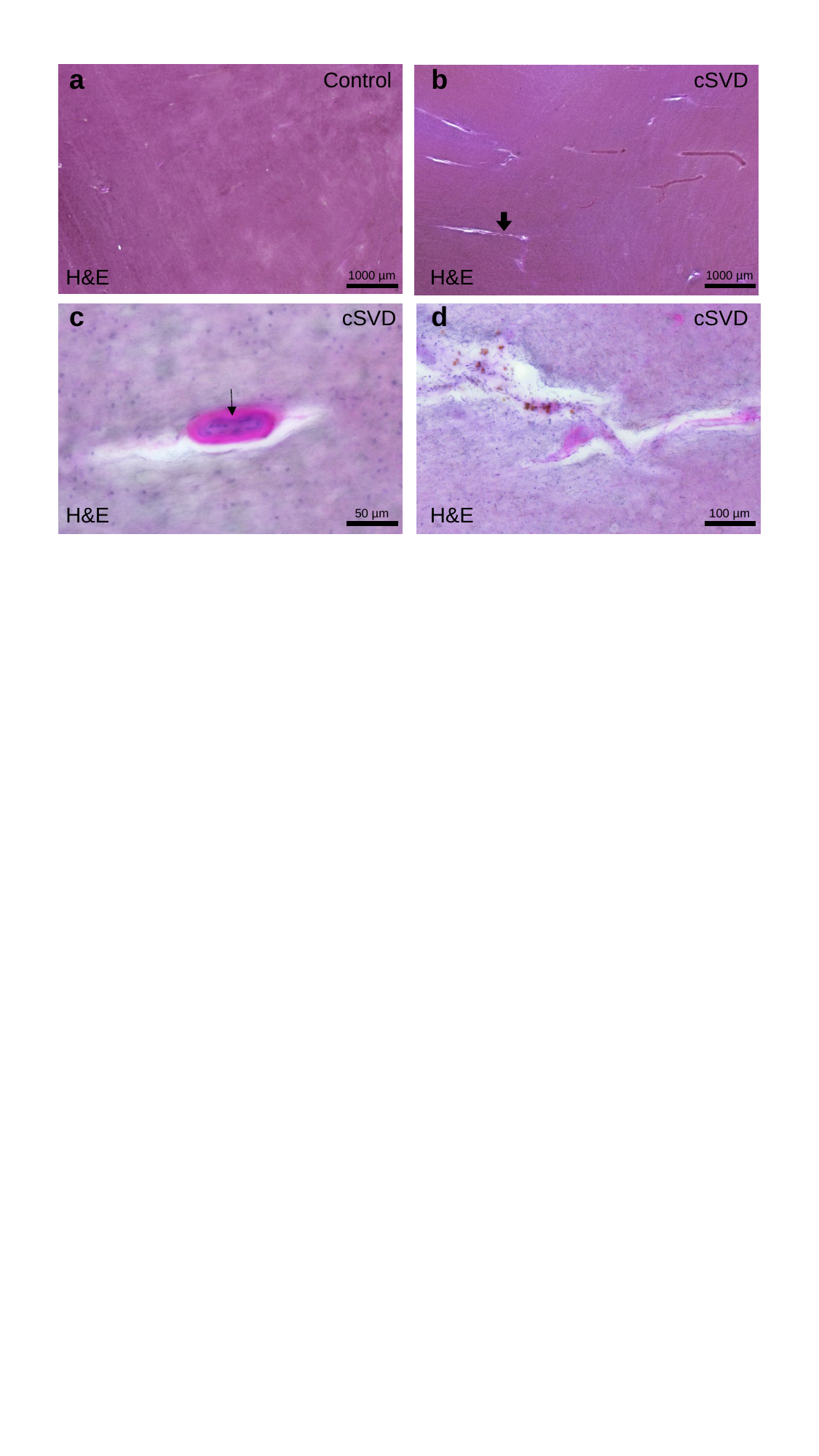

a
b
Control
cSVD
H&E
H&E
1000 µm
1000 µm
c
d
cSVD
cSVD
H&E
H&E
50 µm
100 µm

## Slide 2
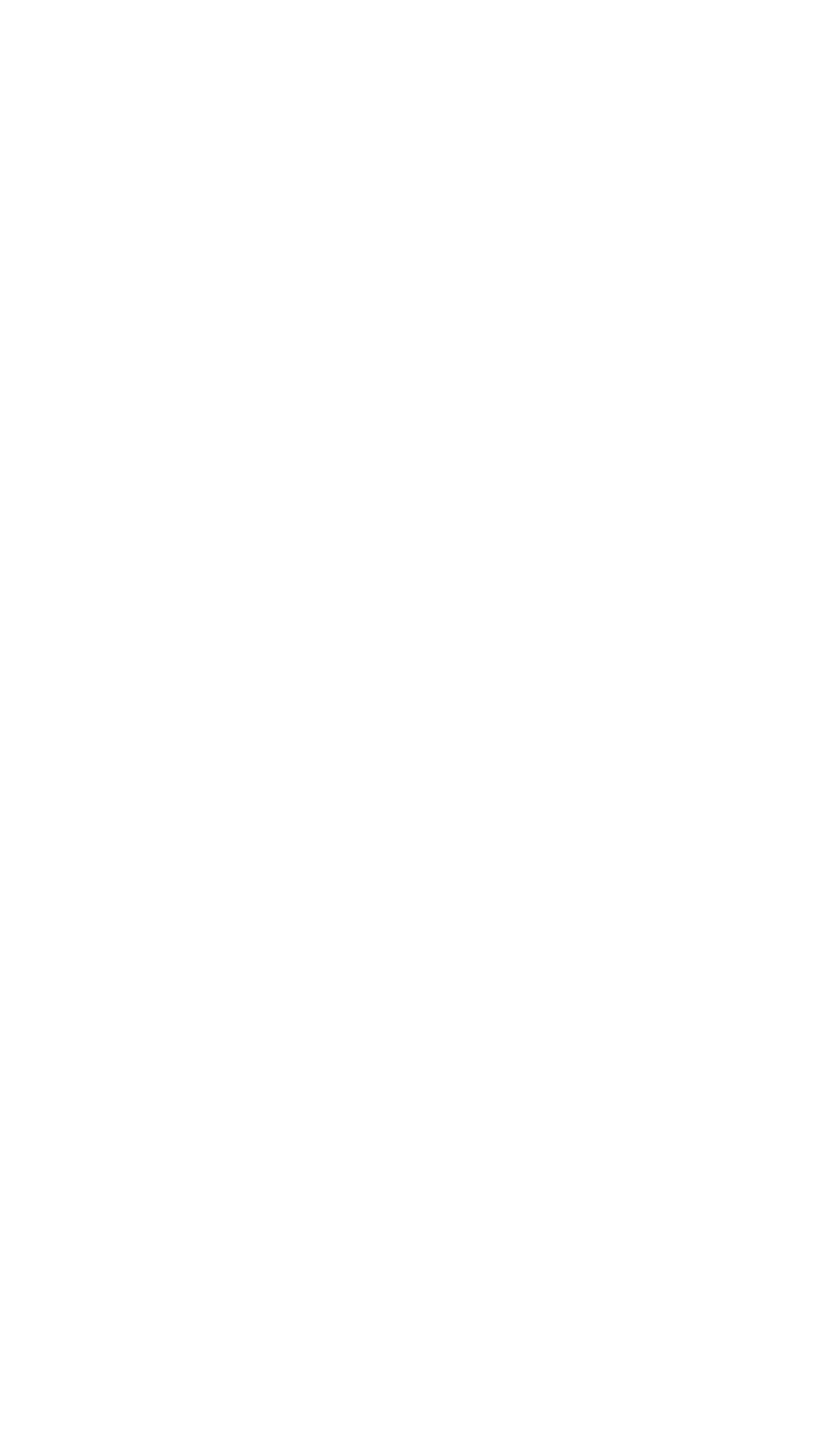

Supplement: Supplementary file 2 — Additional file 2: Fig. S1. Pathological hallmarks of arteriosclerosis (AS) in the human brain visualized in the subcortical white matter with the modified H&E stain in 100µm-thick brain sections. In comparison with control cases (a), overview images show tortuous vessels running in enlarged perivascular spaces (thick black arrow) in the white matter cSVD cases (b). (c) White matter vessel with hyalinosis in the tunica media (thin black arrow). (d) Aggregates of brown hemosiderin in the perivascular space of a white matter vessel indicative of old microbleed (PPTX 4330 kb) [file 40478_2023_1672_MOESM2_ESM.pptx]

a

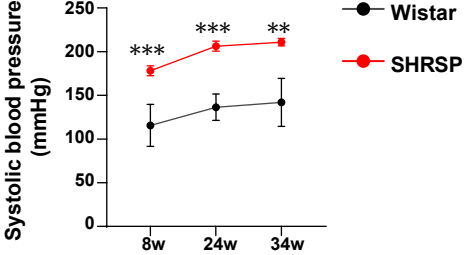

Supplement: Supplementary file 3 — Additional file 3: Fig. S2. SHRSP exhibit elevated systolic blood pressure from the age of 8-weeks onwards compared to age-matched Wistar controls. Systolic blood pressure was measured in Wistar rats and SHRSP (n = 3 per group) at 8, 24 and 34 weeks of age by indirect tail-cuff method. Pressure and pulse rate signals were continuously recorded and digitalized using BP-2000 Analysis Software (BP-2000 Analysis System, 4-channels, Visitech Systems, Apex, NC, USA). Systolic blood pressure was determined as the mean of ten cuff inflation measurements. Data are represented as mean ± SEM. Statistical analysis was performed using 2way ANOVA with Holm-Sidák’s post hoc test for group and age comparison. p-values: ** for p ≤ 0.01; *** for p ≤ 0.001 (PDF 139 kb) [file 40478_2023_1672_MOESM3_ESM.pdf]

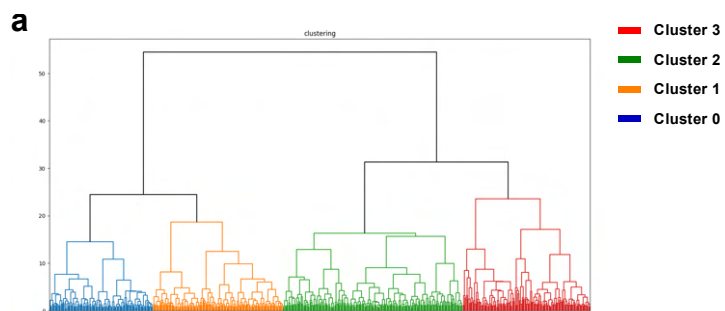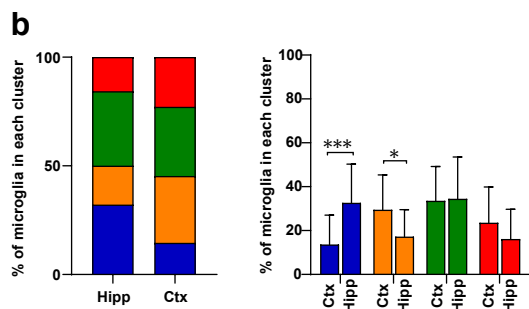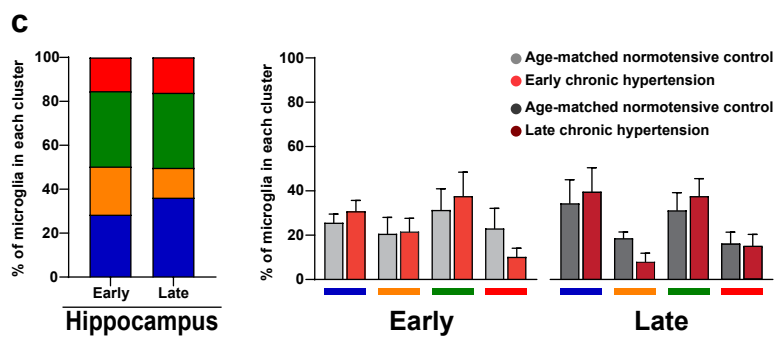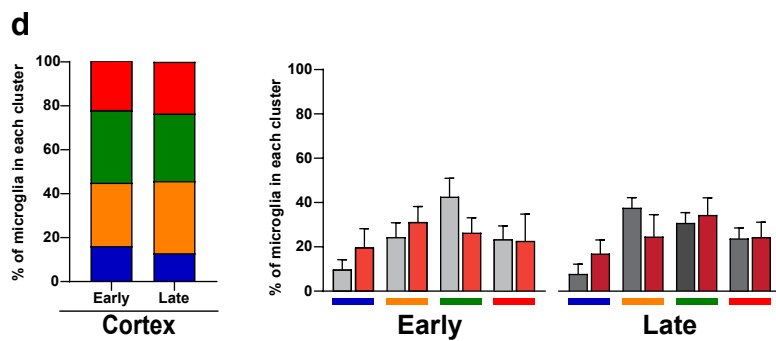

Supplement: Supplementary file 4 — Additional file 4: Fig. S3. Hippocampal and cortex microglia distribution based on 8 morphological features. (a) Ward hierarchical clustering dendrogram of a total of 600 individual Iba1+ cells representative of hippocampal CA1 region and retrosplenial cortex captured for individual 3D reconstruction and used in further analysis. (b) Relative frequencies of microglia categorized into four distinct morphological clusters in the hippocampus and cortex based on subregions as categorical value. (c) Relative frequencies of microglia in the hippocampus and (d) the cortex at early and late chronic hypertension. Statistical analysis was performed using 2way ANOVA with Holm-Sidák’s post hoc test for region and cluster comparison. p-values: * ≤ 0.05; *** for p ≤ 0.001 (PDF 194 kb) [file 40478_2023_1672_MOESM4_ESM.pdf]

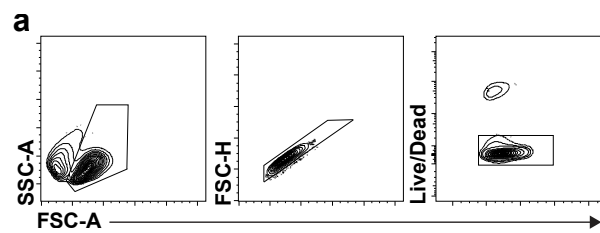

## Hippocampus

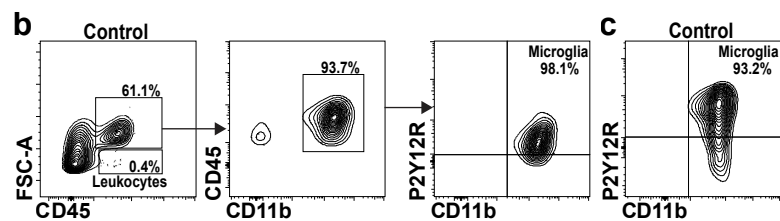

## Cortex

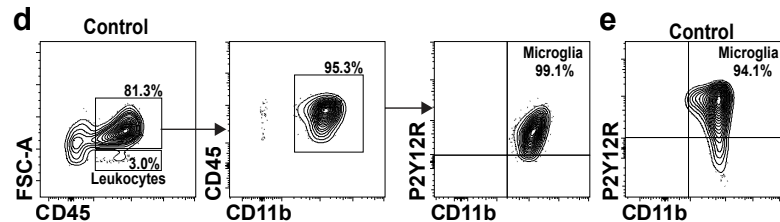

Supplement: Supplementary file 5 — Additional file 5: Fig. S4. Initial gating strategy and identification of Microglia in normotensive hippocampus and cortex. (a) Representative flow cytometric analysis of isolated cells derived from normotensive and hypertensive brains. Cells were selected according to their size and granularity in the forward (FSC-A) and side light scatters (SSC-A). Thereafter, single cells were selected in regard to the ratio of their cell size vs. cell signal displayed in the FSC-H/FSC-A plot. Finally, dead cells were identified by their high affinity to live/dead dye resulting in a brighter fluorescence than live cells. Only live cells were selected for further analysis. (b, c) Identification of microglia derived from the hippocampus and (d, e) cortex of normotensive controls via FACS analysis (PDF 313 kb) [file 40478_2023_1672_MOESM5_ESM.pdf]

## Hippocampus

### Early hypertension

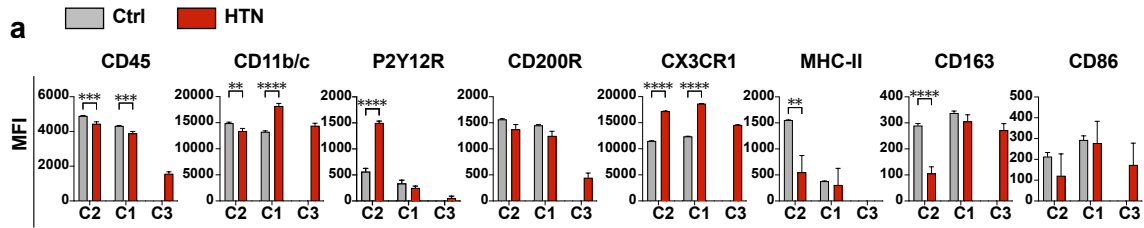

### Late hypertension

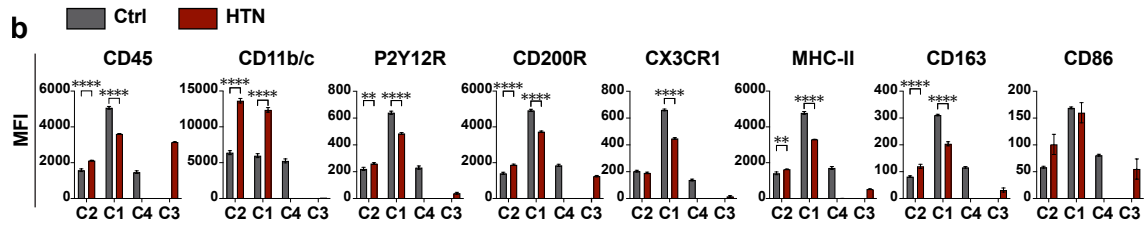

Supplement: Supplementary file 6 — Additional file 6: Fig. S5. Chronic hypertension results in microglia dynamical changes in the hippocampus of hypertensive rats. Bar charts showing the median fluorescence intensity (MFI) of each surface antigen investigated in each hippocampal microglia cluster in early (a) and chronic hypertensive stages (b). Bar charts represent mean ± SEM. Ctrl, Controls; HTN, Hypertension. p-values: ** for p ≤ 0.01; *** for p ≤ 0.001; **** for p ≤ 0.0001 (PDF 146 kb) [file 40478_2023_1672_MOESM6_ESM.pdf]

## Early hypertension

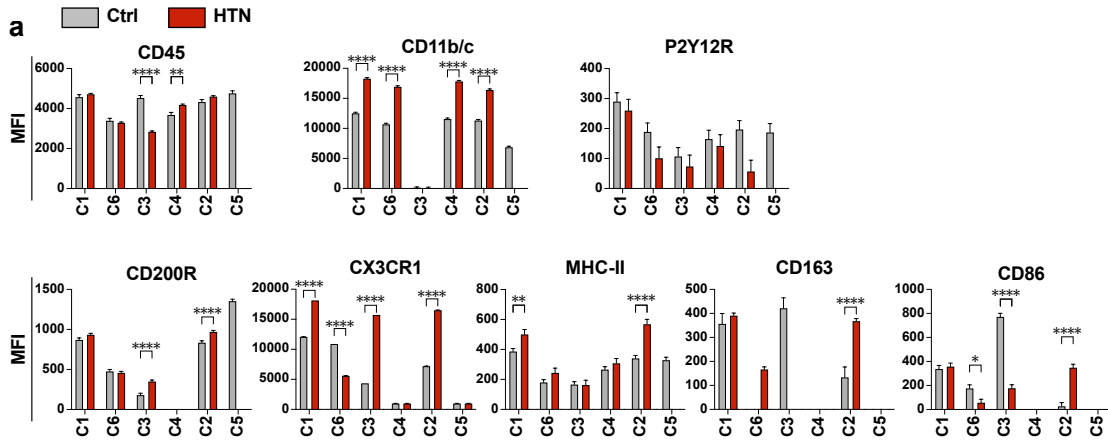

## Late hypertension

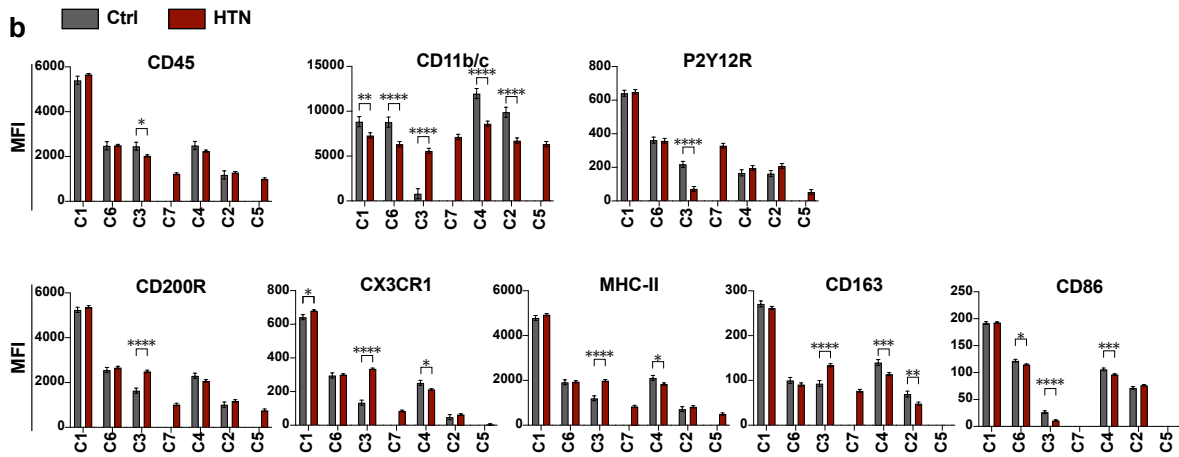

Supplement: Supplementary file 7 — Additional file 7: Fig. S6. Chronic hypertension results in microglia dynamical changes in the cortex of hypertensive rats. Bar charts showing the median fluorescence intensity (MFI) of each surface antigen investigated in each cortical microglia cluster in early (a) and chronic hypertensive stages (b). Bar charts represent mean ± SEM. Ctrl, Controls; HTN, Hypertension. p-values: * ≤ 0.05; ** for p ≤ 0.01; *** for p ≤ 0.001; **** for p ≤ 0.0001 (PDF 150 kb) [file 40478_2023_1672_MOESM7_ESM.pdf]

Cortex

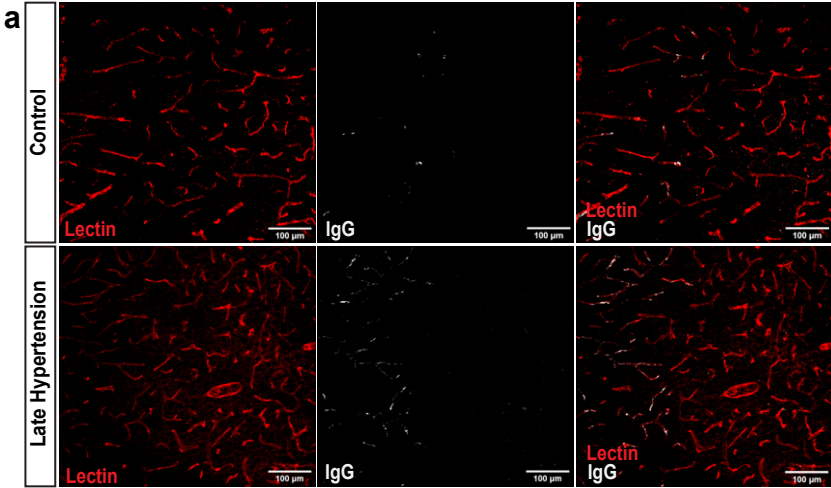

Hippocampus

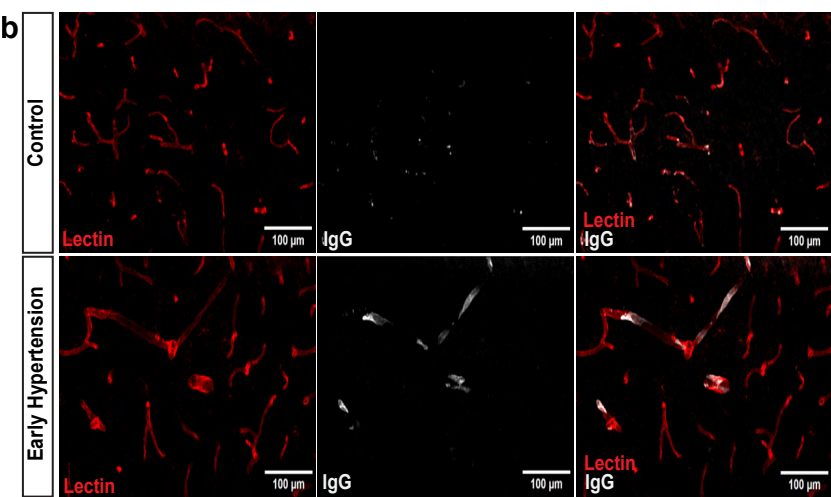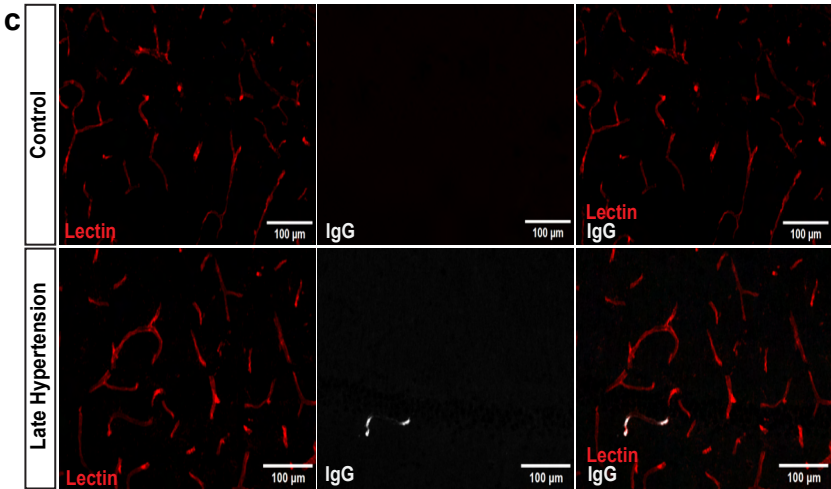

Supplement: Supplementary file 8 — Additional file 8: Fig. S7. Comparative IgG fluorescence in cortical and hippocampal brain regions in chronic hypertensive states. (a) Representative IgG Fluorescence analyzed in cortical brain sections in late chronic hypertension and in hippocampal CA1 region in early (b) and late (c) chronic hypertensive stages. IgG, immunoglobulin G (PDF 685 kb) [file 40478_2023_1672_MOESM8_ESM.pdf]
